# Ratiometric Boltzmann thermometry with Cr^3+^ in strong ligand fields: Efficient nonradiative coupling for record dynamic working ranges

**DOI:** 10.1038/s41377-025-02082-8

**Published:** 2025-11-25

**Authors:** Gülsüm Kinik, Ingo Widmann, Benedikt Bendel, Hubert Huppertz, Andries Meijerink, Markus Suta

**Affiliations:** 1 Inorganic Photoactive Materials, Institute of Inorganic Chemistry, Heinrich Heine University Düsseldorf, Düsseldorf, Germany; 2 Department of General, Inorganic and Theoretical Chemistry, University of Innsbruck, Innsbruck, Austria; 3 Condensed Matter & Interfaces, Debye Institute for Nanomaterials Science, Utrecht University, Utrecht, The Netherlands

**Keywords:** Inorganic LEDs, Optical sensors

## Abstract

A new ratiometric Boltzmann thermometry approach is presented for the narrow-line red-emitting bright phosphor Al_0.993_Cr_0.007_B_4_O_6_N. It relies on thermalization between the two excited states ^2^*E*_*g*_(^2^G) and ^2^*T*_1*g*_(^2^G) of Cr^3+^ with an energy gap of 620 cm^−1^ for optimized thermometry at room temperature. It is shown that nonradiative coupling between these excited states is very fast, with rates in the order of several µs^−1^. Due to the comparably slow radiative decay (*k*_r_ = 0.033 ms^−^^1^) of the lowest excited ^2^*E*_*g*_(^2^G) state, the dynamic working range of this Boltzmann thermometer for the deep red spectral range is exceptionally wide, between <77 K and >873 K, even outperforming the classic workhorse example of Er^3+^. At temperatures above 340 K, also spectrally well-resolved broad-band emission due to the spin-allowed ^4^*T*_2*g*_(^4^F) → ^4^*A*_2*g*_(^4^F) transition is detectable, which simultaneously offers a possibility of very sensitive (*S*_r_(500 K) > 2% K^−1^) ratiometric Boltzmann-type crossover thermometry for higher temperatures. These findings imply that Al_0.993_Cr_0.007_B_4_O_6_N is a particularly robust and bright red luminescent thermometer with a record-breaking dynamic working range for a luminescent transition metal ion.

## Introduction

Remote luminescence thermometers gained increasing attraction for applications in biomedical, physical, and technological fields in the last two decades^1,2^. Within that time, the field matured, and not only has it now been established how to specifically optimize the performance of luminescent thermometers, but also which artifacts can arise that may introduce systematic errors^2–5^. Various application areas have been explored for this technique, such as catalysis^6^, flow velocimetry^6^, or more selective applications as intracellular imaging^7,8^ or measurements of heat transfer at the nanoscale^9–12^. Among the various possibilities to perform luminescence thermometry, the purpose of narrow-line emission from two thermally coupled levels has become particularly attractive^13^. The intensity ratio of such luminescent thermometers obeys Boltzmann’s law and thus allows a simple and accurate temperature control with clear physical input^14^. The major representative emitters for this class of luminescence thermometers are the trivalent lanthanoids (Ln^3+^) with their rich 4f^*n*^ energy level structure throughout the ultraviolet (UV), visible, and near-infrared spectral range^13,15^. Trivalent lanthanoids have many energy levels with definite splittings in the order of a few *k*_B_*T* for temperature ranges varying by the respective splitting energies and have characteristic narrow 4f^*n*^-4f^*n*^ (*n* = 2 for Pr^3+^ to *n* = 13 for Yb^3+^) emission lines for accurate ratiometric luminescence thermometry.

Despite their promising features for this application area, trivalent lanthanoid ions generally suffer from small absorption cross sections of their 4f^*n*^ ↔ 4f^*n*^ transitions (10^−20^ to 10^−21^ cm^2^)^16,17^ that limits their overall brightness. This can pose problems for the precision of luminescent thermometers. Transition metal ions with d^*n*^-d^*n*^ transitions usually show higher absorption cross sections (10^−19^ to 10^−20 ^cm^2^)^18–21^ for a wide optical range^22^ than their lanthanoid congeners. In addition, the broad-band nature of the d^*n*^-d^*n*^ transitions of transition metal ions additionally enhances the brightness as the integrated absorption cross section can then get enhanced by a factor of 10^2^ to 10^3^ compared to lanthanoid ions. However, it is difficult to transfer the appealing concept of Boltzmann thermometry using narrow-line emission from two thermally coupled excited levels to luminescent transition metal ions, as these typically show broad emission bands. This makes it difficult to accurately determine intensity ratios and the luminescence is also prone to thermal quenching by nonradiative crossover^23,24^. The exploitation of vibronic fine structure (Stokes and anti-Stokes lines) in the case of narrow-line emitting Mn^4+^ is a possible alternative that allows for combining stronger absorption strengths with narrow-line emission^25^. In addition, ratiometric thermometry is promising for the 3d^3^ ion Cr^3+^ in strong ligand fields using narrow line emission from the two excited states ^2^*E*_*g*_(^2^G) and ^2^*T*_1*g*_(^2^G) in an octahedral ligand field. Absorption at higher energies can occur upon one-electron excitation into the more antibonding *e*_*g*_-type orbitals under spin conservation, thus giving rise to broad bands with higher absorption cross sections (and thus brightness) than for the trivalent lanthanoid ions.

A well-known representative for this type of phosphor is ruby, *α*-Al_2_O_3_:Cr^3+^. While it shows intense ^4^*T*_2(*g*)_(^4^F) ← ^4^*A*_2(*g*)_(^4^F)-based absorption in the green range, giving rise to its deep red color, the emission spectrum of ruby is dominated by two narrow ^2^*E*_(*g*)_(^2^G) → ^4^*A*_2(*g*)_(^4^F)-based so-called *R* lines. The two *R* lines arise from radiative transitions out of the two Kramers’ doublets $\bar{\text{E}}$ and $2\bar{\text{A}}$ as a consequence of weak spin-orbit coupling and the slight trigonal distortion at the Al sites in corundum-type *α*-Al_2_O_3_, allowing for luminescence thermometry at cryogenic temperatures by exploitation of the small energy gap (Δ*E* = 29 cm^−1^) between the two Kramers’ doublets^26^.

Robust ratiometric Boltzmann thermometry with Cr^3+^ at around room temperature would be possible by thermal coupling between the two excited spin-flip states ^2^*E*_(*g*)_(^2^G) and ^2^*T*_1(*g*)_(^2^G) with a mutual energy gap of 540 cm^−1^ for ruby and 620 cm^−1^ for AlB_4_O_6_N:Cr^3+^ (*T*_opt_ ≈ 250 K–430 K)^27^. Both narrow-line transitions to the ground level are only visible in very strong ligand fields, in which no crossover between the ^2^*E*_(*g*)_(^2^G) and ^4^*T*_2(*g*)_(^4^F) states interferes that gives rise to a broad background due to the spin-allowed ^4^*T*_2(*g*)_(^4^F) → ^4^*A*_2(*g*)_(^4^F) emission. A strong crystal field also raises the luminescence quenching temperature of the ^2^*E*_(*g*)_ and ^2^*T*_1*g*_(^2^G) emissions. While the energy gap of the two thermally coupled excited levels determines the optimum temperature range (*T*_opt_), the excited state dynamics have a strong impact on its dynamic working range. Only if the nonradiative transition rates (which increase with temperature) between the two levels become faster than the radiative decay rate from either of the two excited states, is a Boltzmann behavior of the luminescence intensity ratio (LIR) to be expected^14,28^. Conversely, analysis of the dynamic working range of a luminescence thermometer can be a useful tool for a better understanding of the intrinsic nonradiative coupling strength between excited states. Apart from the well-known energy-gap law stating that the intrinsic nonradiative transition rate becomes exponentially damped with an increasing number *p* of effective vibrational modes^29,30^, there are not many other factors known to have a significant impact on the nonradiative coupling strength. This is in strong contrast to radiative transitions, for which mechanisms for lifting selection rules, control over the local photonic density of states, and cavity quantum electrodynamics have been established for a long time already^29,31–33^. In the case of lanthanoid ions, some efforts have been made to theoretically describe nonradiative transitions in a similar way to radiative transitions by e.g. van Dijk and Schuurmans^34,35^, Orlovskii and Pukhov^36–38^as well as Macfarlane^39^. They demonstrated that multi-phonon nonradiative relaxation rates could also be described in an analogous framework to Judd-Ofelt theory, as they are proportional to the oscillator strength of the 4f^*n*^-4f^*n*^ transition involved in the lanthanoid ion that transfers the energy to a vibrational overtone *via* Förster-type dipole-dipole interaction^40^. In line with those findings, some of us recently showed that this can have a significant impact on the dynamic working range of lanthanoid ions as luminescent Boltzmann thermometers^41^. In contrast, selection rules for nonradiative relaxation in organic emitters are much more established^42–44^, as internal (spin-allowed) conversion between excited spin singlet states is usually in the order of ps, while (spin-forbidden) intersystem crossing to excited spin triplet states typically occurs in the order of ns to µs. In addition to that, El-Sayed’s rules allow for estimating when intersystem crossing is expectedly to be faster depending on the change in the contributing orbital nature of the related excited states^45^.

**Fig. 1:**
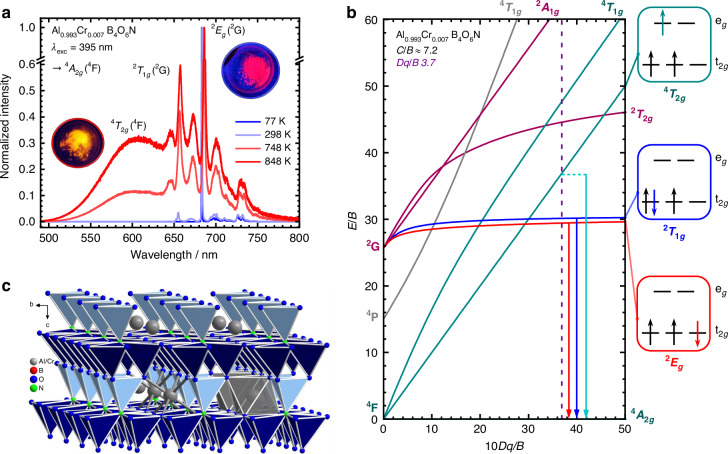
Optical properties of octahedrally coordinated Cr^3+^ ions in AlB_4_O_6_N. **a** Representative emission spectra of Al_0.993_Cr_0.007_B_4_O_6_N at selected temperatures depicting both narrow-line ^2^*T*_1*g*_, ^2^*E*_*g*_(^2^G) → ^4^*A*_2*g*_(^4^F)- and broad-band ^4^*T*_2*g*_(^4^F) → ^4^*A*_2*g*_(^4^F)-based emission (break at *y* axis for better visualization of both emission bands). **b** Effective Tanabe-Sugano diagram based on estimated Racah parameters *B* and *C* for Al_0.993_Cr_0.007_B_4_O_6_N. The vertical dashed line depicts the *Dq*/*B* ratio for Al_0.993_Cr_0.007_B_4_O_6_N. The d^3^ micro-configurations in an octahedral crystal field related to the multielectron states are depicted on the right side of each diagram with the respective emission denoted as a colored arrow. **c** Structure of Al_0.993_Cr_0.007_B_4_O_6_N with view along the crystallographic *a* axis emphasizing the octahedral coordination of the Al^3+^/Cr^3+^ ions (in grey) embedded in a condensed network of [BO_3_N]^6-^ tetrahedra (O atoms in blue, N atoms in green)

Recently, we reported about Al_0.97_Cr_0.03_B_4_O_6_N as an analog of ruby with an even stronger ligand field for incorporated Cr^3+^ ions than in *α*-Al_2_O_3_^27^. Its structure is homeotypic to swedenborgite (NaBe_4_SbO_7_^46^) and the nitridosilicate BaYbSi_4_N_7_^47^ with Al sites that are almost perfectly octahedrally coordinated by six O^2-^ ions^27,48^. This structural feature gives rise to the observation of a single *R* line since the high symmetry does not split the ^2^*E*_*g*_(^2^G) level even under the action of spin-orbit coupling, in contrast to ruby. The high symmetry of the Al sites in AlB_4_O_6_N provides an ideal test ground for selection rules of multi-phonon type nonradiative transitions for states that sensitively react to the surrounding ligand field. It was thus the purpose of this work to explicitly compare the thermometric performance of the ^2^*T*_1(*g*)_(^2^G)–^2^*E*_(*g*)_(^2^G) gap of Cr^3+^ in Al_0.993_Cr_0.007_B_4_O_6_N (see Fig. 1a) and *α*-Al_2_O_3_:Cr^3+^ offering a robust ratiometric thermometry concept with a transition metal ion.

## Results

### Structural and photoluminescence properties of Al_0.993_Cr_0.007_B_4_O_6_N

The photoluminescence of AlB_4_O_6_N activated with 0.7 mol% Cr^3+^ indicates its potential as an alternative remote optical pressure calibration standard to ruby (for diffraction patterns and structural data, see Figs. S1–S3 and Tab. S1)^27^. CrB_4_O_6_N has been reported to have a light red body color^49^, while powdered Al_0.993_Cr_0.007_B_4_O_6_N is colorless. The low concentration of Cr^3+^ is necessary to avoid quenching that usually occurs in phosphors at higher activator fractions and thus affects the decay kinetics of the relevant excited states investigated within this work. Al_0.993_Cr_0.007_B_4_O_6_N shows red narrow-line luminescence based on the ^2^*E*_*g*_(^2^G) → ^4^*A*_2*g*_(^4^F) upon excitation at 365 nm, similar to ruby (see Fig. 1a). Upon temperature increase, however, a broad emission band related to the ^4^*T*_2*g*_(^4^F) → ^4^*A*_2*g*_(^4^F) transition of Cr^3+^ can be detected. Figure 1b depicts the Tanabe-Sugano diagrams for a d^3^ ion using the estimated Racah and ligand field parameters for Al_0.993_Cr_0.007_B_4_O_6_N. Additional factors compared to *α*-Al_2_O_3_:Cr^3+^ as the absorption cross section for the excitation source, pressure stability, the temperature-dependent shift, and the relative sensitivity were discussed by some of us before^27^. Both compounds contain octahedrally coordinated Al^3+^ sites with a low distortion leading to a lowered site symmetry of *C*_3*v*_. These are favored for the incorporation of Cr^3+^ ions in 3d^3^ configuration. The average Al–O bond lengths for the Al site (see the gray colored octahedron in Fig. 1c) in AlB_4_O_6_N (1.89 Å) and *α*-Al_2_O_3_:Cr^3+^ (1.91 Å), based on single-crystal data matching with the spectrally resolved transition energies of Al_0.993_Cr_0.007_B_4_O_6_N and *α*-Al_2_O_3_ (<1 ppm Cr^3+^) in the ligand field approach by the angular overlap model, suggest a slightly stronger ligand field acting on the 3d orbitals of the incorporated Cr^3+^ ions in AlB_4_O_6_N, which is beneficial for a high energy of the excited ^4^*T*_2*g*_(^4^F) state^27^.

In addition, the PLE spectra of AlB_4_O_6_N reveal the presence of the broad-band ^4^*T*_2*g*_(^4^F) ← ^4^*A*_2*g*_(^4^F) transition at 510 nm and ^4^*T*_1*g*_(^4^F) ← ^4^*A*_2*g*_(^4^F) transition at 393 nm (see Fig. S4). The emission spectrum is dominated by a strong narrow zero-phonon line related to the ^2^*E*_*g*_(^2^G) → ^4^*A*_2*g*_(^4^F) transition over the entire temperature course (see Fig. 1a), which does not split any further in an octahedral ligand field under the action of spin-orbit coupling as the ^2^*E*_*g*_(^2^G) state retains its total degeneracy of 2 (spin degeneracy) x 2 (orbital degeneracy) = 4 (see also Figs. S5 for Al_0.993_Cr_0.007_B_4_O_6_N and S6 for *α*-Al_1.993_Cr_0.007_O_3_).

### Ratiometric Boltzmann-type luminescence thermometry in Al_0.993_Cr_0.007_B_4_O_6_N

**Fig. 2:**
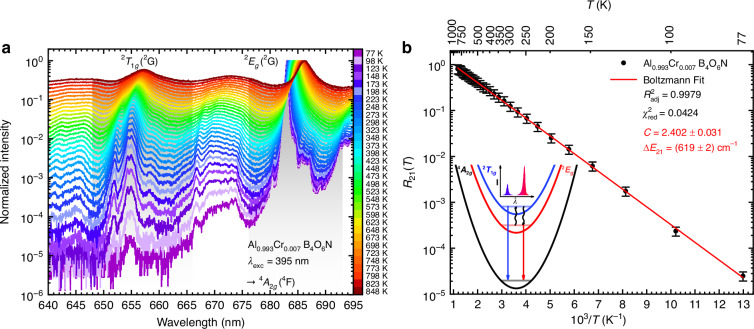
Boltzmann-type luminescence thermometry with Cr^3+^ in AlB_4_O_6_N. **a** Temperature-dependent normalized emission spectra of Al_0.993_Cr_0.007_B_4_O_6_N in a semi-logarithmic scale. The integration ranges for the ^2^*T*_1*g*_(^2^G) → ^4^*A*_2*g*_(^4^F) and ^2^*E*_*g*_(^2^G) → ^4^*A*_2*g*_(^4^F) transitions are highlighted as grey areas, respectively. **b** Plot of the temperature-dependent LIR *R*_21_(*T*) between the integrated intensities of the ^2^*T*_1*g*_(^2^G) → ^4^*A*_2*g*_(^4^F) and ^2^*E*_*g*_(^2^G) → ^4^*A*_2*g*_(^4^F)-based emission lines against the inverse temperature. The red lines are the least-squares fit to Eq. 1. The errors in **b** were estimated assuming Poissonian photon counting statistics of the photomultiplier

The experimental details for photoluminescence measurements of this work are provided in the Supplementary Information (Section S1.2). Figure 2a depicts the temperature-dependent emission spectra of Al_0.993_Cr_0.007_B_4_O_6_N from 77 K to 848 K, which is compared with the temperature-dependent luminescence of classic ruby (*α*-Al_1.993_Cr_0.007_O_3_, Figs. S7 and S8). In both cases, dominant narrow-line, spin-forbidden emission from the ^2^*E*_*g*_(^2^G) state is observed with a temperature-induced red-shift due to the thermal expansion and a consequently increasing Cr–O bond length, while the line broadening is based on stronger electron-phonon coupling (see Figs. 1a and 2a)^27,50^. Another transition based on the emission from the ^2^*T*_1*g*_(^2^G) state is also detectable at around 655 nm, which must be a consequence of thermalization with the ^2^*E*_*g*_(^2^G) state. Since both the ^2^*E*_*g*_(^2^G) → ^4^*A*_2*g*_(^4^F) and ^2^*T*_1*g*_(^2^G) → ^4^*A*_2*g*_(^4^F) transitions are sufficiently narrow (FWHM < 30 cm^−1^), an energy gap of 614 cm^−1^ can be spectrally resolved – a situation that is otherwise typically only encountered for the trivalent lanthanoid ions with their narrow-line 4f^*n*^ ↔ 4f^*n*^ transitions. The LIR of the two observed radiative transitions indeed shows a clear Boltzmann-type behavior down to at least 77 K according to^51^

1 $${R}_{21}\left(T\right)=\frac{{I}_{20}}{{I}_{10}}=C\frac{{g}_{2}}{{g}_{1}}\exp \left(-\frac{\Delta {E}_{21}}{{k}_{{\rm{B}}}T}\right)$$

with *g*_2_ = 6 and *g*_1_ = 4 as degeneracies of the ^2^*T*_1*g*_(^2^G) = |2〉 and ^2^*E*_*g*_(^2^G) = |1〉 states, *C* = *k*_2r_/*k*_1r_ as the electronic preexponential factor representing the ratio of radiative decay constants of the two excited states, Δ*E*_21_ as the energy gap, and *k*_B_ as the Boltzmann constant. The fit of the experimentally observed intensity ratios to Eq. 1 (ref. ^51^) in Fig. 2b shows an excellent agreement over the full temperature range investigated (77 – 850 K) with an energy difference Δ*E*_21_ of (619 ± 2) cm^−1^ (see Figs. S5c, d and Table S4). This finding indicates an extraordinarily large dynamic working range for Al_0.993_Cr_0.007_B_4_O_6_N as a luminescent thermometer. That type of ratiometric thermometry with Cr^3+^ has so far been scarcely considered, given the relatively rare scenario of a sufficiently strong ligand field in most inorganic compounds. A representative example was reported by Heinze et al. in the case of the complex [Cr(ddpd)_2_]^3+^ (ddpd = *N*,*N*′-dimethyl-*N*,*N*′-dipyridin-2-ylpyridine-2,6-diamine) known as “molecular ruby”^52^. Instead, the majority of reported works on luminescence thermometry with Cr^3+^ rely on the thermal crossover to the excited ^4^*T*_2*g*_(^4^F) state that gives rise to broad-band emission of Cr^3+^ ^53–57^. In the case of *α-*Al_2_O_3_:Cr^3+^, also ratiometric cryothermometry exploiting the small energy gap (Δ*E*_21_ = 29 cm^−1^) between the two Kramers’ doublets $2\bar{{\rm{A}}}$ (Γ_5,6_) and $\bar{{\rm{E}}}$ (Γ_4_) has been reported^55,58^. Finally, lifetime-based thermometry approaches were investigated both for Cr^3+^ and the isoelectronic Mn^4+^ ion^59–65^. Thermal occupation of the ^4^*T*_2*g*_(^4^F) excited state with a spin-allowed transition to the ground state gives rise to pronounced shortening of the luminescence decay time in a temperature range depending on the ^2^*E*_*g*_(^2^G) – ^4^*T*_2*g*_(^4^F) energy gap.

A limitation to the presented new thermometric approach with Cr^3+^ at high temperatures is thermal crossover to the excited ^4^*T*_2*g*_(^4^F) state (see also Fig. 1b). Both in Al_0.993_Cr_0.007_B_4_O_6_N and *α*-Al_2_O_3_:Cr^3+^, this quenching pathway only becomes significant at temperatures above *T*_1/2_ = 570 K (Al_0.993_Cr_0.007_B_4_O_6_N) and *T*_1/2_ = 463 K (*α*-Al_2_O_3_: < 1ppm Cr^3+^) (see Fig. S9). The higher quenching temperature of Al_0.993_Cr_0.007_B_4_O_6_N than in *α*-Al_2_O_3_:Cr^3+^ can be related to the stronger ligand field splitting 10*Dq* acting on the 3d orbitals of the Cr^3+^ activator in AlB_4_O_6_N. As a result of the large crystal field strength, the ^4^*T*_2*g*_(^4^F) excited state is at a very high energy, even much higher than in ruby, which makes it possible to observe both ^2^*E*_*g*_(^2^G) and ^2^*T*_1*g*_(^2^G) narrow-line emission up to very high temperatures and can explain the record high quenching temperature for the Cr^3+^ emission. Overall, the use of the ^2^*T*_1*g*_(^2^G)- and ^2^*E*_*g*_(^2^G)-based emission lines in Al_0.993_Cr_0.007_B_4_O_6_N constitutes an example of a particularly robust ratiometric thermometer with a record dynamic working range between at least 77 K and 850 K.

Given the energy gap of Δ*E*_21_ = (619 ± 2) cm^−1^ between the ^2^*E*_*g*_(^2^G) and ^2^*T*_1*g*_(^2^G) state in Al_0.993_Cr_0.007_B_4_O_6_N, the optimum temperature range *T*_opt_ defined by

2 $${T}_{\mathrm{opt}}\in \left[\frac{\Delta {E}_{21}}{\left(2+\sqrt{2}\right){k}_{{\rm{B}}}},\,\frac{\Delta {E}_{21}}{{2k}_{{\rm{B}}}}\right]$$

is *T*_opt_ ∈ [260 K, 445 K]^14^. The energy gap also matches the spectroscopically determined value from excitation spectra of 614 cm^−1^ at 20 K (see Fig. S4 and Table S3). Thus, ratiometric thermometry with the two radiative narrow-line transitions ^2^*T*_1*g*_(^2^G) → ^4^*A*_2*g*_(^4^F) and ^2^*E*_*g*_(^2^G) → ^4^*A*_2*g*_(^4^F) is suited for thermodynamically optimized, precise temperature sensing in a wide range, including room temperature. Within *T*_opt_, the relative sensitivity,

3 $${S}_{{\rm{r}}}\left(T\right)=\frac{\Delta {E}_{21}}{{k}_{{\rm{B}}}{T}^{2}}$$

 with all symbols as defined above, varies from *S*_r_(260 K) = 1.32% K^−1^ to *S*_r_(445 K) = 0.45% K^−1^ for Al_0.993_Cr_0.007_B_4_O_6_N (see Fig. S10a). However, it should be stressed that the relative sensitivity alone does not determine the overall statistical precision of a ratiometric luminescent thermometer, but brightness is as important^3^. If photons are detected with a photon counting system, the relative statistical uncertainty of a ratiometric luminescent thermometer is given by^14^

4 $$\frac{{\sigma }_{T}}{T}=\frac{{k}_{{\rm{B}}}T}{\Delta {E}_{21}}\frac{1}{\sqrt{{I}_{10}}}\sqrt{1+\frac{1}{{R}_{21}\left(T\right)}}$$

with *I*_10_ as a given intensity of the lower energetic (typically more intense) emission, in this case the ^2^*E*_*g*_(^2^G) → ^4^*A*_2*g*_(^4^F)-based emission, and *R*_21_(*T*) as the temperature-dependent LIR. The temperature-dependent evolution of the relative statistical uncertainty of the presented thermometry concept with Cr^3+^ is schematically depicted in Fig. S10b for exemplary values of *I*_10_. If the integrated intensity of the ^2^*E*_*g*_(^2^G) → ^4^*A*_2*g*_(^4^F) exceeds 10^6^ counts, theoretically expected minimum relative temperature readout uncertainties *σ*_*T*_/*T* close to 0.1% are feasible within *T*_opt_ with Cr^3+^-activated AlB_4_O_6_N. In fact, this thermometer performs better than classic *α*-Al_1.993_Cr_0.007_O_3_ (see Table 1 and Fig. S10). Cycling experiments for selected LIRs also demonstrate that both Al_0.993_Cr_0.007_B_4_O_6_N and *α*-Al_1.993_Cr_0.007_O_3_ work as reproducible, robust thermometers (see Figs. S11–S13).

**Table 1:** Relevant parameters for the thermometric performance of narrow-line emitting Al_0.993_Cr_0.007_B_4_O_6_N (see Fig. 2) and *α*-Al_1.993_Cr_0.007_O_3_ (see Fig. S7)

	Al_0.993_Cr_0.007_B_4_O_6_N		*α*−Al_1.993_Cr_0.007_O_3_
*T*_on_		<77 K	<50 K^a^
∆*E*_21_		(619 ± 2) cm^−1^	(532 ± 7) cm^−^^1^
*S*_r_(300 K)		0.99% K^−1^	0.85% K^−1^
*k*_1r_		0.030 ms^−1^	0.189 ms^−1^
*T*_opt_		[260 K, 445 K]	[225 K, 380 K]
*T*_1/2_		570 K^b^	463 K^b^

^a^based on Eq. 5 with literature-reported *k*_nr_(0) from transient absorption measurements^66^

^b^see Fig. S9

For more explanations, see text

### Characterization of the nonradiative transition between the ^2^*T*_1*g*_(^2^G) and ^2^*E*_*g*_(^2^G) level in Cr^3+^-activated AlB_4_O_6_N and *α*-Al_2_O_3_

At sufficiently low temperatures, the LIR of Cr^3+^-activated AlB_4_O_6_N is expected to deviate from Boltzmann behavior^14,28^. This deviation can be related to the decoupling of the ^2^*T*_1*g*_(^2^G) and ^2^*E*_*g*_(^2^G) states and offers information about the mutual intrinsic nonradiative coupling strength, *k*_nr_(0), which determines the dynamic working range of a luminescent thermometer. At low temperatures, the nonradiative absorption rate, ${k}_{\text{nr}}^{\text{abs}}(T)$, from the lower excited state |1〉 to the higher excited one |2〉 may not be competitive to the total decay rate from the lower excited level |1〉, *k*_1_ = *k*_1r_ + *k*_quench_ = *k*_1r_/*ϕ*_1_(0) (with *ϕ*_1_(0) as the internal quantum yield from level |1〉), and thermal equilibrium between the two excited states cannot be sustained anymore. For resonant bridging of the two excited states by one vibrational quantum, this equilibrium leads to a kinetically defined onset temperature (*T*_on_) for thermalization between the two excited states of^41^

5 $${T}_{\mathrm{on}}=\frac{\Delta {E}_{21}}{{k}_{{\rm{B}}}{\text{ln}}\left[1+\,\frac{{g}_{2}{k}_{\mathrm{nr}}(0)}{{k}_{1{\rm{r}}}}\right]}$$

with Δ*E*_21_ as the energy gap between the two excited states, *k*_B_ as the Boltzmann constant, *g*_2_ as the degeneracy of the higher energetic excited state |2〉 (here: *g*_2_ = 6), and *k*_nr_(0) as the intrinsic nonradiative transition rate constant. Formally, any deviation of the LIR from Boltzmann behavior or mutual deviation of the luminescent decay times of the two coupled excited states at sufficiently low temperatures can give an indication of the previously mentioned decoupling of the two excited states. Detection of the time-resolved luminescence from both the ^2^*T*_1*g*_(^2^G) and ^2^*E*_*g*_(^2^G) state reveal similar decay times within statistical significance down to 80 K (see Fig. S14). This implies thermal coupling of the two excited states even at that low temperature. Additional confirmation can be gained from an estimate of the luminescence intensity of the ^2^*T*_1*g*_(^2^G) → ^4^*A*_2*g*_(^4^F)-related emission according to Boltzmann’s law (*R*_21_(77 K) ≈ 1.52 ∙ 10^−5^ based on Eq. 1), in close agreement to the experimentally estimated (height-based) LIR of 1.57 ∙ 10^−5^ (see Fig. 2a) at 77 K. Overall, the thermal coupling between the ^2^*T*_1*g*_(^2^G) and ^2^*E*_*g*_(^2^G) states in Al_0.993_Cr_0.007_B_4_O_6_N must be very fast and allows a rough estimate *k*_nr_(0) > 1 µs^−1^ according to Eq. 5. It was not possible to derive a more accurate value as the determination relies on the measurable intensity of the ^2^*T*_1*g*_(^2^G) → ^4^*A*_2*g*_(^4^F)-based emission, which has a very low signal-to-noise ratio below 77 K. We can, however, anticipate that the value of *k*_nr_(0) = 1 µs^−1^ in Al_0.993_Cr_0.007_B_4_O_6_N is still severely underestimated, as transient absorption studies on ruby at liq. He temperatures (4.2 K) revealed a corresponding nonradiative transition rate of *k*_nr_(0) = (400 ± 80) µs^−1^ ^66^, while Hartree-Fock calculations even led to higher estimates (*k*_nr_(0) ≈ 8 ps^−1^)^56,67^. Future pump-probe or photon echo experiments on Al_0.993_Cr_0.007_B_4_O_6_N may help find a more accurate estimate of the nonradiative coupling rate constant *k*_nr_(0) for the ^2^*T*_1*g*_(^2^G) → ^2^*E*_*g*_(^2^G) transition and compare it to the rates known for the trivalent lanthanoid ions^34,35^. That concept of selection rules for nonradiative transitions is well-established for intersystem crossing in molecular emitters^68,69^ and has been recently indicated for the lanthanoid ions by Burshtein^70^, but is so far lacking a more general picture. Assuming a similarly high value of *k*_nr_(0) ≈ 400 µs^−1^ as a nonradiative transition rate for the ^2^*T*_1*g*_(^2^G) → ^2^*E*_*g*_(^2^G) transition in Al_0.993_Cr_0.007_B_4_O_6_N, we estimate a kinetic *T*_on_ of 50 K according to Eq. 5. It should be noted, however, that at these temperatures the emission intensity from the ^2^*T*_1*g*_(^2^G) level is so weak that thermometry is practically not feasible in that temperature range.

It is interesting to compare the presented thermometry approach with the commonly used thermally coupled excited, green-emitting levels ^2^H_11/2_ and ^4^S_3/2_ (Δ*E*_21_ ≈ 650 cm^−1^) of Er^3+^ in inorganic upconversion (nano-) phosphors such as *β*-NaYF_4_:Er^3+^, Yb^3+^ ^71,72^. The energy gap Δ*E*_21_ is similar. The main advantage of the Cr^3+^-based thermometer is higher brightness because of the much stronger absorption for the spin-allowed absorption bands. Additionally, the internal photoluminescence quantum yield is generally higher for narrow-line emitting Cr^3+^ than for the green-emitting levels of Er^3+^ since they are prone to additional nonradiative relaxation to lower energetic ^4^F_9/2_ levels and also emission from the thermally coupled ^2^H_11/2_ and ^4^S_3/2_ levels to other 4f^11^ levels than the ^4^I_15/2_ ground state lowers the brightness for the emission used for LIR thermometry. Another example of robust background-free thermometry at room temperature was demonstrated with the ^6^P_5/2_ and ^6^P_7/2_ levels of Gd^3+^ (again with a similar energy gap Δ*E*_21_ ≈ 600 cm^−1^) that can be excited by blue light in an upconversion mechanism *via* an energy transfer from Pr^3+^ in YAl_3_(BO_3_)_4_^73^. This mechanism involves the excited 4f^1^5d^1^ configuration of Pr^3+^ in the UV range. Thus, thermal coupling between the ^2^*T*_1*g*_(^2^G) and ^2^*E*_*g*_(^2^G) states of Cr^3+^ in AlB_4_O_6_N or *α*-Al_2_O_3_ constitutes a third example of a robust luminescent Boltzmann thermometer with the highest expected precision at around room temperature.

**Fig. 3:**
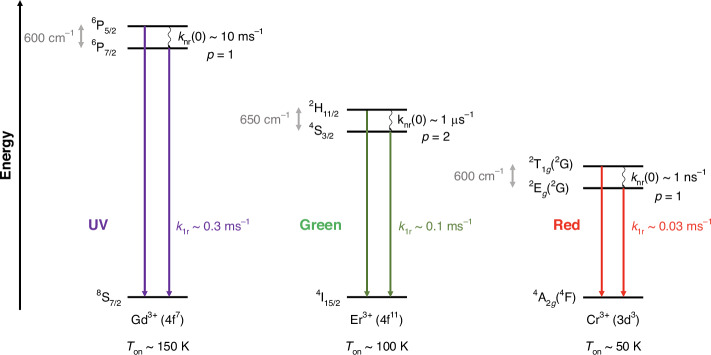
Comparison of the performance of UV-emitting Gd^3+^ (left), green-emitting Er^3+^ (middle), and red-emitting Cr^3+^ (right) as ratiometric Boltzmann thermometers^51,74–77^. Typical radiative and intrinsic nonradiative transition rates are indicated, which result in the given sequence of *T*_on_ for thermalization of the excited states

In order to better understand the underlying reason for the differences in performance as luminescent thermometers, it is insightful to analyze the excited state dynamics of the previously mentioned emitters (see Fig. 3). In *β*-NaYF_4_:Er^3+^, Yb^3+^, the radiative decay rate of the lower energetic ^4^S_3/2_ level of the Er^3+^ ions is around *k*_1_ ~ 1.6 ms^−1^ ^74^. The nonradiative transition between the two excited ^2^H_11/2_ and ^4^S_3/2_ levels of Er^3+^ has a high probability because of the strong induced electric dipolar character, which is indicated by the relatively large values of the reduced matrix elements ${{||}\left\langle {U}^{\left(k\right)}\right\rangle {||}}^{2}\left({||}{\left\langle {U}^{\left(2\right)}\right\rangle {||}}^{2}=0.0000,{{||}\left\langle {U}^{\left(4\right)}\right\rangle {||}}^{2}=0.2002,{{||}\left\langle {U}^{(6)}\right\rangle {||}}^{2}=0.0097\right)$ for the ^2^H_11/2_ ↔ ^4^S_3/2_ transition^75^. Consequently, the nonradiative transition is orders of magnitude faster (*k*_nr_(0) ~ 1 µs^−1^) than radiative decay from the ^4^S_3/2_ level, resulting in low *T*_on_ for thermal coupling of around 100 K (see Fig. 3) in *β*-NaYF_4_: 2% Er^3+^, 18% Yb^3+^ ^76^ or YVO_4_: 0.1% Er^3+^ ^77^.

In contrast, the *T*_on_ of UV-emitting Gd^3+^ ions exploiting a resonant one-phonon transition in complex oxide-based hosts such as vanadates, phosphates, or borates to nonradiatively bridge the energy gap between the excited ^6^P_5/2_ and ^6^P_7/2_ levels (Δ*E*_21_ ≈ 600 cm^−1^) is found to be higher (*T*_on_ ≈ 150 K, see Fig. 3) since both the radiative decay from the lower energetic ^6^P_7/2_ level and the nonradiative transition between these two excited levels of Gd^3+^ have strong magnetic dipolar character and are intrinsically slow. However, while the respective nonradiative coupling strength is much smaller (*k*_nr_(0)~10 ms^−1^)^51^ compared to the anticipated value for Cr^3+^ in AlB_4_O_6_N, the radiative decay of the corresponding ^6^P_7/2_ → ^8^S_7/2_-based emission (*λ*_em_~310 nm) of Gd^3+^ is much faster (*k*_1_~0.3 ms^−1^) than for the ^2^*E*_*g*_(^2^G) → ^4^*A*_2*g*_(^4^F)-based emission of Cr^3+^ in AlB_4_O_6_N. The observed onset temperature *T*_on_ for thermalization of excited states depends on the ratio of the nonradiative absorption and radiative decay rate (see Eq. 5). In Al_0.993_Cr_0.007_B_4_O_6_N, the unusually strong ligand field and high local site symmetry of the Al sites together with the very effective nonradiative coupling strength between the ^2^*T*_1*g*_(^2^G) and ^2^*E*_*g*_(^2^G) states leads to a hitherto unrealized wide dynamic working range of a luminescent Boltzmann thermometer based on Cr^3+^. Moreover, the broad-band and the related higher oscillator strengths for the spin-allowed absorption transitions compared to the trivalent lanthanoid ions together with the high internal quantum yield of the ^2^*E*_*g*_(^2^G)-based radiative emission ensures a very high brightness, which is key to a high statistical precision of a luminescent thermometer^2,3^. Thus, next to a UV-emitting luminescent Boltzmann thermometer based on Gd^3+^ ^73^ and a green-emitting Boltzmann thermometer based on Er^3+^ ^71,72^, thermal coupling between the ^2^*T*_1(*g*)_(^2^G) and ^2^*E*_(*g*)_(^2^G) states of Cr^3+^ in AlB_4_O_6_N or *α*-Al_2_O_3_ constitutes a third example (see Fig. 3) of a robust ratiometric Boltzmann thermometer with highest expected precision in the range around room temperature and offers clear advantages over the other thermometers: emission in the deep red to near-infrared range (deeper penetration depth in biological samples), higher brightness (stronger absorption) and a wider dynamic temperature range as a result of faster nonradiative relaxation between thermally coupled states.

The underlying reason for the so much stronger nonradiative coupling between the ^2^*T*_1*g*_(^2^G) and ^2^*E*_*g*_(^2^G) states of Cr^3+^ compared to the coupling between the 4f^*n*^ spin-orbit levels of the trivalent lanthanoid ions must be related to the lateral extension of the outer 3d orbitals compared to the shielded inner 4f orbitals and the resulting higher electron-phonon coupling strength of the former orbitals. The stronger electron-phonon coupling is also reflected in the much stronger vibronic transitions observed for ^2^*E*_*g*_(^2^G) → ^4^*A*_2*g*_(^4^F) emission of Cr^3+^ compared to that for 4f^*n*^-4f^*n*^ transitions of lanthanide ions^78–86^. Related theoretical ideas going towards this direction were reported by Kushida and Kikuchi^67^. Selection rules do not appear to play a significant role here as a symmetry analysis would imply a magnetic dipolar and thus, expectedly slow nonradiative ^2^*T*_1*g*_(^2^G) ↔ ^2^*E*_*g*_(^2^G) transition. Overall, the ratiometric thermometry concept exploiting the two narrow emission lines based on the ^2^*T*_1*g*_(^2^G) → ^4^*A*_2*g*_(^4^F) and ^2^*E*_*g*_(^2^G) → ^4^*A*_2*g*_(^4^F) transitions of Cr^3+^ in AlB_4_O_6_N together with the very high thermal quenching temperature due to the unusually strong ligand field leads to an unprecedented dynamic working range of a red-emitting transition metal-based activator.

### Nonradiative crossover between the ^4^*T*_2*g*_(^4^F) and ^2^*E*_*g*_(^2^G) states in Cr^3+^-activated AlB_4_O_6_N

**Fig. 4:**
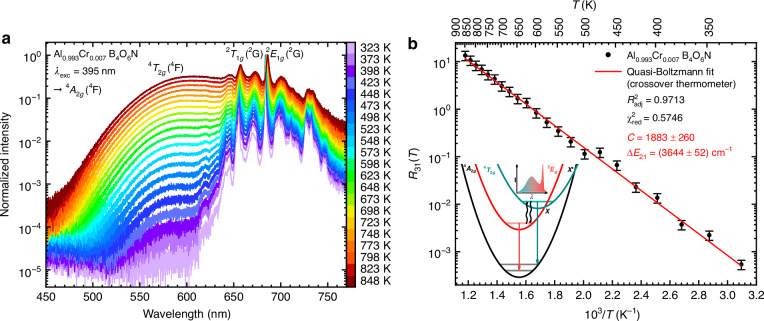
Thermalization between the ^2^E_g_(^2^G) and ^4^T_2g_(^4^F) states in Al_0.993_Cr_0.007_B_4_O_6_N with potential for high-temperature luminescence thermometry. **a** Temperature-dependent emission spectra of Al_0.993_Cr_0.007_B_4_O_6_N between 323 K and 848 K. The integration ranges for the ^4^*T*_2*g*_(^4^F) → ^4^*A*_2*g*_(^4^F) and ^2^*E*_*g*_(^2^G) → ^4^*A*_2*g*_(^4^F) transition were varied due to the band-broadening and the red-shift of the emission bands by temperature, respectively. For absolute intensity spectra, see Fig. S16. **b** LIR *R*_31_(*T*) between the integrated intensities of the ^4^*T*_2*g*_(^4^F) → ^4^*A*_2*g*_(^4^F)- and ^2^*E*_*g*_(^2^G) → ^4^*A*_2*g*_(^4^F)-based emission bands in Al_0.993_Cr_0.007_B_4_O_6_N with the least-squares fit to Eq. 7 with *A* → 0

In both Al_0.993_Cr_0.007_B_4_O_6_N and *α*-Al_1.993_Cr_0.007_O_3_, an additional broad emission band is observed above ~300 K or 200 K, respectively, which is assigned to the ^4^*T*_2*g*_(^4^F) → ^4^*A*_2*g*_(^4^F) transition (see Fig. 4 as well as Fig. S8). Up to 850 K, the intensity of this emission band increases accompanied by a simultaneous decrease of the narrow-line ^2^*T*_1*g*_(^2^G), ^2^*E*_*g*_(^2^G) → ^4^*A*_2*g*_(^4^F)-based emission. The observation of the broad-band emission and related thermalization between the ^2^*E*_*g*_(^2^G) and ^4^*T*_2*g*_(^4^F) states of Cr^3+^ is in excellent agreement with the modeled temperature dependence of the luminescence decay times with thermally averaged decay times of 2.5 ms at 873 K for Al_0.993_Cr_0.007_B_4_O_6_N and 675 µs at 598 K for *α*-Al_2_O_3_:Cr^3+^ (see Fig. S15). The luminescence decay times for Cr^3+^ in AlB_4_O_6_N are longer because of the high symmetry (closer to inversion symmetry than for Cr^3+^ in ruby) and start to decrease at higher temperatures related to the stronger ligand field in Al_0.993_Cr_0.007_B_4_O_6_N, which shifts the ^4^*T*_2*g*_ level to higher energies compared to ruby, giving rise to a larger energy gap between the ^2^*E*_*g*_ and ^4^*T*_2*g*_ states. Accordingly, the temperature-dependent LIR between the ^4^*T*_2*g*_(^4^F) → ^4^*A*_2*g*_(^4^F)- and ^2^*T*_1*g*_(^2^G), ^2^*E*_*g*_(^2^G) → ^4^*A*_2*g*_(^4^G)-based emission bands follows a Boltzmann behavior at sufficiently high temperature, which can be derived from the analytic steady-state solution,^87,88^

6 $${R}_{31}\left(T\right)=\frac{{I}_{30}}{{I}_{10}}=\frac{{k}_{3{\rm{r}}}}{{k}_{1{\rm{r}}}}\frac{{\alpha }_{a3}{k}_{1{\rm{r}}}+{g}_{3}{k}_{\mathrm{nr}}\left(0\right)\exp \left(-\frac{\Delta {E}_{X1}}{{k}_{{\rm{B}}}T}\right)}{(1-{\alpha }_{a3}){k}_{3{\rm{r}}}+{g}_{1}{k}_{\mathrm{nr}}\left(0\right)\exp \left(-\frac{\Delta {E}_{3X}}{{k}_{{\rm{B}}}T}\right)}$$

with *α*_a3_ the feeding ratio from the pumped ^4^*T*_1*g*_(^4^F) = |*a*〉 state to the *g*_3_ = 12-fold degenerate ^4^*T*_2*g*_(^4^F) = |3〉 state. It is a reasonable assumption that the nonradiative relaxation from the ^4^*T*_1*g*_(^4^F) to the energetically close ^4^*T*_2*g*_(^4^F) state is much faster than to the energetically lower *g*_1_ = 4-fold degenerated ^2^*E*_*g*_(^2^G) = |1〉 state (especially since the latter also involves a spin-flip) which means that *α*_a3_ ≈ 1. Δ*E*_*X*1_ denotes the barrier between the vibrational ground level of the ^2^*E*_*g*_(^2^G) state to the crossover point *X* with the ^4^*T*_2*g*_(^4^F) potential energy curve, while Δ*E*_3*X*_ is the respective (lower) barrier for the reverse crossover from the vibrational ground state of the ^4^*T*_2*g*_(^4^F) state to *X*.

Under the assumption of *α*_a3_ ≈ 1, Eq. 6 can be simplified to the more useful fitting equation

7 $$\begin{array}{rcl}{R}_{31}\left(T\right)=\frac{{I}_{30}}{{I}_{10}}\!\!\!&=&\!\!\!\frac{1}{{g}_{1}{\beta }_{3}}\exp \left(\frac{\Delta {E}_{3X}}{{k}_{{\rm{B}}}T}\right)+\,\frac{{g}_{3}}{{g}_{1}}\frac{{\beta }_{3}}{{\beta }_{1}}\exp \left(-\frac{\Delta {E}_{31}}{{k}_{{\rm{B}}}T}\right)\\ &&\!\!\!\!\!\!\!\!\!\!\!\!\!\!\!\!\!\!\!\!\!\!\!\!\!\!\! \approx A+C\frac{{g}_{3}}{{g}_{1}}\exp \left(-\frac{\Delta {E}_{31}}{{k}_{{\rm{B}}}T}\right)\end{array}$$

with *β*_1,3_ = *k*_nr_(0)/*k*_1,3r_ and Δ*E*_31_ = Δ*E*_*X*1_ – Δ*E*_3*X*_ as the vertical (0–0) energy gap between the ^4^*T*_2*g*_(^4^F) and ^2^*E*_*g*_(^2^G) state^89^. Since Δ*E*_3*X*_ is only a small barrier (typically ≤ 500 cm^−1^), it can be estimated that *A* < 0.1 at temperatures above 77 K and is thus negligible for the regarded temperature range of *T* > 320 K (see Fig. 4, A(*T* = 320 K) ≈ 10^−5^ for Δ*E*_3*X*_ ~ 500 cm^−1^). It should be noted that approximation (6) may imply an unusual increase of the ^4^*T*_2*g*_(^4^F)-based emission in the limit Δ*E*_3*X*_ ≳ *k*_B_*T* (i.e., for very low temperatures), which is certainly not observed (see Fig. S4) and indicates that Δ*E*_3*X*_ must be negligibly small.

Common radiative decay rate constants *k*_3r_ of the ^4^*T*_2*g*_(^4^F) state in e.g., Cr^3+^-activated garnets are in the order of *k*_3r_ ≈ 10^2^ – 10^3 ^ms^−1^ (refs.^49,60,79–83,90–94^). From the temperature-dependent decay measurements (see Fig. S9), a value of *k*_3r_ = *αk*_1r_ = (161 ± 20) ms^−1^ is estimated for Al_0.993_Cr_0.007_B_4_O_6_N, while the probed decay of the ^4^*T*_2*g*_(^4^F)-based emission at 873 K (see Fig. S15 and Eq. S4) allows an estimate of *k*_3r_ = (36 ± 5) ms^−1^ exactly in the reported range mentioned above^60,90–94^. Additional confidence about the right order of magnitude can be gained from Herzberg’s perturbative mixing approach of electronic states^93,95,96^ (see Eqs. S2 and S3), which yields an estimate of *k*_3r_ ≈ 60 ms^−1^.

With the knowledge of the radiative decay rate *k*_3r_ of the ^4^*T*_2*g*_(^4^F) state of Cr^3+^ in AlB_4_O_6_N, it is possible to gain physical insight into the parameters *A* and *B* obtained from fitting Eq. (7) to the temperature-dependent LIR *R*_31_(*T*) (see Fig. 4). The value of *C* = *k*_3r_/*k*_1r_ is expected to be 10^3^ – 10^4^, in excellent agreement with the fitted value (*C* = 1.88 ∙ 10^3^). In contrast, *A* is dominated by the ratio between *k*_3r_ and *k*_nr_(0) and thus, expectedly much smaller than 1. In fact, the parameter *A* cannot be experimentally accurately determined given the very low relative intensity of the ^4^*T*_2*g*_(^4^F) → ^4^*A*_2*g*_(^4^F)-based emission in the range of 10^-5^ counts at temperatures below 320 K indicate that nonradiative relaxation from ^4^*T*_2*g*_ to ^2^*T*_1*g*_ and ^2^*E*_*g*_ is so much faster than radiative decay from the ^4^*T*_2*g*_ level that no ^4^*T*_2*g*_ emission is observed at low temperatures. Both values are, however, in the expected range. The fit of the temperature-dependent LIR to Eq. 7 (see Fig. 4b) yields an energy gap of Δ*E*_31_ = (3644 ± 52) cm^−1^, which agrees very well with the estimated energy gap between the ^4^*T*_2*g*_(^4^F) and ^2^*E*_*g*_(^2^G) states from the temperature-dependent decay measurements (Δ*E*_31_ ≈ (3837 ± 103) cm^−1^, Fig. S9) and from the energy difference of 3728 cm^−1^ determined from the positions of the ^2^*E*_*g*_ and ^4^*T*_2*g*_ zero-phonon lines at 20 K (see Table S3 and Figs. S4 and S5). Despite this large energy gap between the ^4^*T*_2*g*_(^4^F) and ^2^*E*_*g*_(^2^G) states, the observed relative integrated intensity of the ^4^*T*_2*g*_(^4^F) → ^4^*A*_2*g*_(^4^F)-based emission (5.38 ∙ 10^−4^) at 320 K matches the expected value according to the fit to Eq. 7 in the limit *A* → 0 (*R*_31_(320 K) = 5.51 ∙ 10^−4^), which indicates that even at that comparatively low temperature, there is already thermalization between the ^4^*T*_2*g*_(^4^F) and ^2^*E*_*g*_(^2^G) states. This finding indicates a very high intrinsic nonradiative coupling rate constant *k*_nr_(0). Again, pioneering transient absorption data reported for other Cr^3+^-activated compounds are insightful here and nonradiative rates for the ^4^*T*_2*g*_(^4^F) → ^2^*E*_*g*_(^2^G) transition of the order of 10^1^ – 10^2 ^ns^−1^ were reported (*k*_nr_(0) = 37 ns^−1^ for alexandrite and *k*_nr_(0) = 142 ns^−1^ for ruby)^60,91,97^. These values are about six orders of magnitude faster than the ^4^*T*_2*g*_(^4^F) → ^4^*A*_2*g*_(^4^F) radiative decay rate, which explains why emission cannot be observed at low temperatures, also considering that the ^4^*T*_2*g*_(^4^F) → ^4^*A*_2*g*_(^4^F)-based emission gives rise to a broad-band compared to the sharp ^2^*E*_*g*_(^2^G) → ^4^*A*_2*g*_(^4^F)-based emission.

The huge energy gap of Δ*E*_31_ of around 3700 cm^−1^ might imply a very sensitive luminescence thermometer at high temperatures (see Figs. S12 and S13). However, the relatively low signal-to-noise of the broad-band ^4^*T*_2*g*_(^4^F) → ^4^*A*_2*g*_(^4^F)-based emission and the significant spectral overlap with the narrow-line ^2^*E*_*g*_(^2^G) → ^4^*A*_2*g*_(^4^F)-based emission pose severe limitations to this crossover-based thermometry approach compared to the alternative way of classic ratiometric Boltzmann thermometry with the two narrow ^2^*T*_1*g*_(^2^G)- and ^2^*E*_*g*_(^2^G)-related emission lines demonstrated above. The overall temperature-dependent color change of the luminescence of Al_0.993_Cr_0.007_B_4_O_6_N can be represented in a CIE diagram (see Fig. S17).

## Discussion

A new accurate, wide temperature range, robust, and bright ratiometric luminescence thermometer with Cr^3+^ in an exceptionally strong ligand field is demonstrated. The narrow-line red-emitting phosphor Al_0.993_Cr_0.007_B_4_O_6_N with ^2^*E*_*g*_(^2^G) and ^2^*T*_1*g*_(^2^G) states separated by around 600 cm^−1^ can be exploited for high-precision Boltzmann thermometry based on the temperature-dependent intensity ratio of the narrow emission lines from the two thermally coupled levels.

Luminescence studies at temperatures below room temperature reveal fundamental insights into the relevance of nonradiative coupling between excited states. The nonradiative transition rate between the ^2^*E*_*g*_(^2^G) and ^2^*T*_1*g*_(^2^G) states of Cr^3+^ is very high (estimated in the order of 0.1 – 1 ns^−1^) and is based on a one-phonon transition between potential energy curves with similar equilibrium geometries. This value is much higher than for nonradiative coupling for similar energy gaps between the shielded inner 4f^*n*^ spin-orbit levels of the trivalent lanthanoid ions and can be understood by the larger spatial extension of the outer 3d orbitals. Together with the unique high thermal quenching temperature *T*_1/2_ = 550 K (again explained by the high energy position of the ^4^*T*_2*g*_ state for Cr^3+^ in AlB_4_O_6_N), the narrow emission lines of Al_0.993_Cr_0.007_B_4_O_6_N offer an unprecedented ultra-wide dynamic working range between <77 K to >850 K as a simple, robust, and precise ratiometric luminescent thermometer emitting in the deep red range. Compared to workhorse lanthanoid ion thermometers based on, e.g., Er^3+^, the new Al_0.993_Cr_0.007_B_4_O_6_N material offers a wider temperature range and higher brightness.

Above 340 K, also broad-band emission based on the ^4^*T*_2*g*_(^4^F) → ^4^*A*_2*g*_(^4^F) can be detected. Given the wide energy gap of ~3700 cm^−1^, such a low onset temperature *T*_on_ implies a very high intrinsic nonradiative transition rate (in the order of 10 ns^−1^). The large energy gap implies a high relative sensitivity (*S*_r_(500 K) > 2% K^−1^) at elevated temperatures, but the relatively low signal-to-noise ratio of the broad-band ^4^*T*_2*g*_(^4^F) → ^4^*A*_2*g*_(^4^F)-based emission as well as the necessity for deconvolution of the emission spectra, limits its application for luminescent thermometry at high temperatures. In conclusion, Al_0.993_Cr_0.007_B_4_O_6_N is a bright LIR thermometer with a hitherto record-breaking dynamic working range emitting in the deep red range. It will be challenging (but rewarding) to find other Cr^3+^-doped materials with even stronger ligand fields to outperform this new luminescent thermometer.

## Supplementary information

Supplemental Material
