# Supplementary material for: Ratiometric Boltzmann thermometry with Cr3+ in strong ligand fields: Efficient nonradiative coupling for record dynamic working ranges
Source: Light Sci Appl. 2025 Nov 25;14:388. doi: 10.1038/s41377-025-02082-8 (PMC12645019; doi:10.1038/s41377-025-02082-8)
Supplement: Supplementary file 1 — Supplemental Material [file 41377_2025_2082_MOESM1_ESM.pdf]

Supplementary Information for

Ratiometric Boltzmann thermometry with Cr^{3+} in strong ligand fields: Efficient nonradiative coupling for record dynamic working ranges

Gülsüm Kinik†[1], Ingo Widmann†[2], Benedikt Bendel[1], Hubert Huppertz*[2] Andries Meijerink*[3], and Markus Suta*[1]

[1] *Inorganic Photoactive Materials, Institute of Inorganic Chemistry, Heinrich Heine University Düsseldorf, Universitätsstraße 1, 40225 Düsseldorf, Germany*

[2] *Department of General, Inorganic and Theoretical Chemistry, University of Innsbruck, Innrain 11 80–82, 6020 Innsbruck, Austria*

[3] *Condensed Matter and Interfaces, Debye Institute for Nanomaterials Science, Department of Chemistry, Utrecht University, Princetonplein 1, 3584 CC Utrecht, The Netherlands*

*Corresponding authors: Hubert.Huppertz@uibk.ac.at; a.meijerink@uu.nl; markus.suta@hhu.de

† These authors contributed equally.

Content

1. Materials and methods 2

1.1. Synthesis and characterization 2

1.2. Photoluminescence spectroscopy 9

2. Results 11

2.1. Photoluminescence spectroscopy at 20 K 11

2.2. Ratiometric (quasi-)Boltzmann thermometry in α-$Al_{1.993}Cr_{0.007}O_3$ (ruby) 17

2.2.1. Temperature-dependent time-resolved luminescence in $Al_{0.993}Cr_{0.007}B_4O_6N$ and α-Al_2O_3:Cr^{3+} (< 1ppm Cr^{3+}) 19

2.2.2. Relative sensitivities $S_r(T)$ and temperature uncertainties σ_T/T of $Al_{0.993}Cr_{0.007}B_4O_6N$ and α-$Al_{1.993}Cr_{0.007}O_3$ 21

2.3. Broad-band $^4T_{2(g)}$ (^{4}F) → $^4A_{2g}$ (^{4}F)-based emission in $Al_{0.993}Cr_{0.007}B_4O_6N$ and α-$Al_{1.993}Cr_{0.007}O_3$ 24

3. References 28

1. Materials and methods

1.1. Synthesis and characterization

$Al_{0.993}Cr_{0.007}B_4O_6N$. For the synthesis of $Al_{0.993}Cr_{0.007}B_4O_6N$, the starting materials B_2O_3 (99.9 %, Strem Chemicals), $Al(NO_3)_3{\cdot}9H_2O$ (≥98 %, Sigma Aldrich), Al_2O_3 (99.99 %, Sinochem Hebei), and Cr_2O_3 (99 %, Merck) were weighed in the stoichiometric ratio 6:1:1:0.01 and thoroughly ground in an agate mortar. 0.7 mol% Al^{3+} was substituted by Cr^{3+} to activate the Al-site of AlB_4O_6N. Next, the reaction mixture was transferred into a *h*-BN crucible (Henze Boron Nitride Products AG), closed with a *h*-BN lid and placed within an octahedral pressure cell of an "18/11 multianvil assembly". This pressure cell is positioned at the center of eight beveled tungsten carbide cubes (HA-7%Co, Hawedia). To ensure quasi-hydrostatic pressure conditions, the synthesis was conducted in a Walker-type multianvil press provided by Voggenreiter GmbH. A more detailed description of the experimental setup is provided in the literature[1-3]. During the synthesis of $Al_{0.993}Cr_{0.007}B_4O_6N$, the maximum pressure of 7.0 GPa was reached within 425 min, the maximum temperature of 1350 °C within 7 min and maintained for 10 min before quenching to room temperature. Subsequently, the assembly was decompressed to ambient conditions within 720 min. The resulting reaction product had the appearance of a delimited sphere in the center, surrounded by another solid product, which exhibited a colorless appearance under visible light and red luminescence under 365 nm ultraviolet light. The fraction solid was separated from the rest of the product mixture and analyzed individually by powder diffraction. Since that fraction appeared be dominantly amorphous, it was not appropriate to be further characterized in this work. Therefore, the analyses described later in this work relate to the actual desired product. Further details on the synthesis and structure of AlB_4O_6N and the Cr^{3+}-activated representatives were reported earlier by us[4].

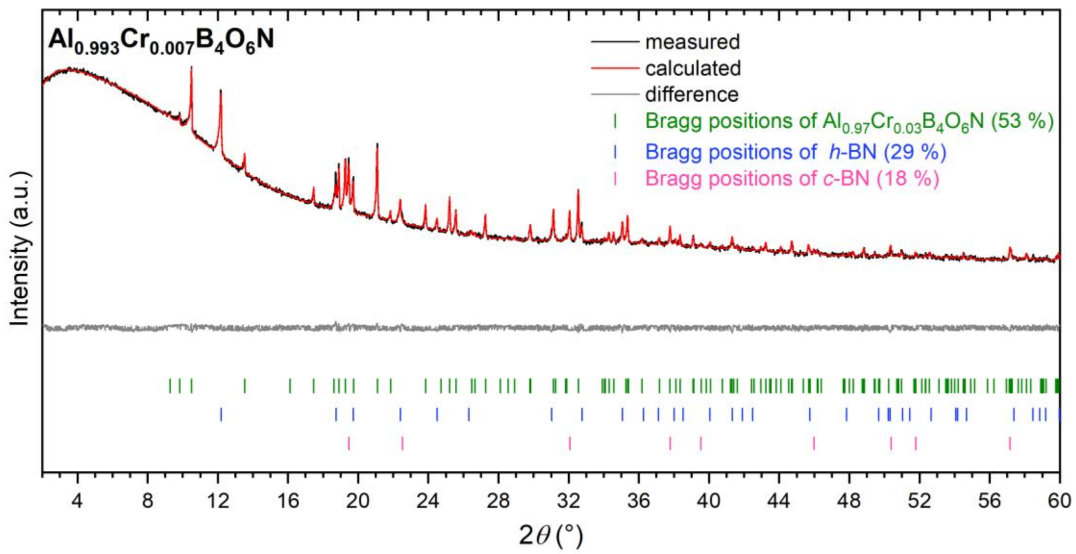

Figure S1. Rietveld refinement of the powder diffraction pattern (Mo$K_{\alpha 1}$ radiation) of the above-described synthesis approach of $Al_{0.993}Cr_{0.007}B_4O_6N$. All assigned phases are specified on the above right corner.

To obtain more product and optimize the synthesis conditions, a second experiment was performed. The starting materials H_3BO_3 (99.8 %, Roth), Al_2O_3 (99.99 %, Sinochem Hebei), *h*-BN (99%, Strem Chemicals), and Cr_2O_3 (99 %, Merck) were weighed in the ratio 5:1:5:0.00076 and thoroughly ground in an agate mortar. The reaction mixture was encapsulated in platinum foil (with a small hole to allow a potential gas exchange) and placed in a crucible made of *h*-BN (Henze Boron Nitride Products AG) and further processed as described above for the other synthesis approach, apart from these synthesis conditions: The maximum pressure of 6.0 GPa was reached within 315 min, the maximum temperature of 1000 °C within 10 min and maintained for 20 min before decreasing the temperature to 700 °C and then quenching to room temperature. Subsequently, the assembly was decompressed to ambient conditions within 720 min. Afterwards, the sample was heated on a crucible formed from a Pt foil in a tube furnace at 500 °C for 1 h (heating rate 300 °C/h) at ambient pressure and under air. The resulting reaction product exhibited a colorless appearance under visible light and showed

intense red luminescence upon excitation with ultraviolet light (λ = 365 nm). According to elemental analysis, the incorporated Cr content was 0.7 mol%.

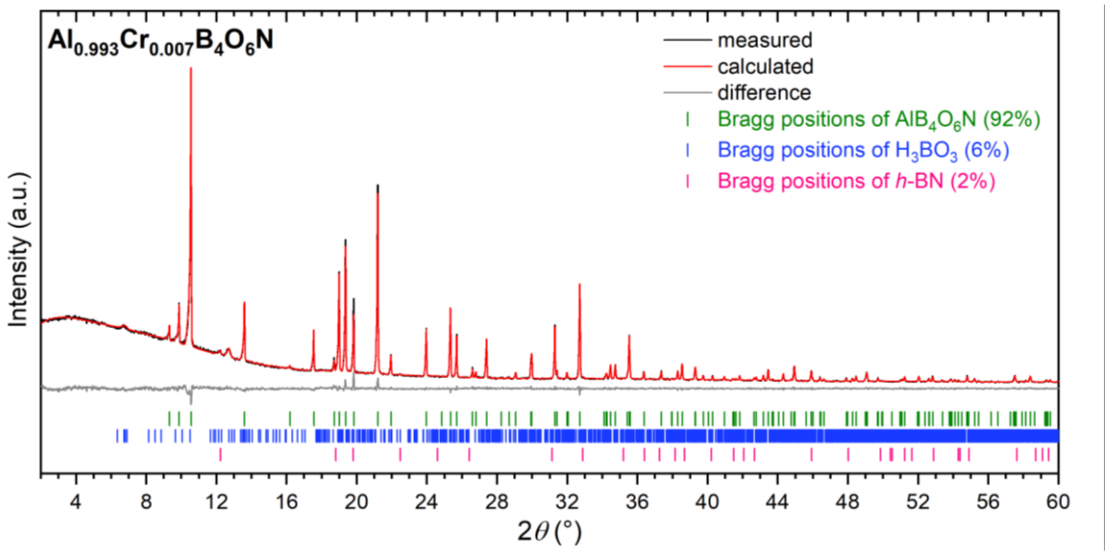

Figure S2. Rietveld refinement of the powder diffraction pattern (Mo$K_{\alpha1}$ radiation) of $Al_{0.993}Cr_{0.007}B_4O_6N$. All assigned phases are specified on the above right corner.

The reaction product was analyzed with a Stoe Stadi P powder diffractometer equipped with a Mythen 1 K microstrip detector (Dectris). The measurement was performed with Ge(111)-monochromatized Mo$K_{\alpha1}$-radiation (λ = 70.93 pm) in transmission geometry across a 2θ range of 2.0 – 60.5°. In order to determine the quantitative amount of $Al_{0.993}Cr_{0.007}B_4O_6N$ and general composition of the thus synthesized powder, the powder X-ray diffraction pattern was further analyzed by means of Rietveld refinement[5] using the TOPAS 4.2[6] software. Additional reflections in the powder X-ray diffraction pattern are related to the presence of *h*-BN[7] and *c*-BN[8], which may both stem from residual parts of the crucible and do not affect the luminescence properties of $Al_{0.993}Cr_{0.007}B_4O_6N$ as the $3d^3$ ion Cr^{3+} is favorably incorporated into an octahedrally coordinated site. In the following sections, the solid solution

$Al_{0.993}Cr_{0.007}B_4O_6N$ from Fig. S1 and S2 is referred to as $Al_{0.993}Cr_{0.007}B_4O_6N$. Both samples were used for the photoluminescence analysis presented in this paper.

α-Al_2O_3:Cr^{3+}. α-Al_2O_3 (ruby) with ≈ (0.35 ± 0.03) mol% activator fractions (according to EDX measurements) of Cr^{3+} was obtained by heating commercial γ-Al_2O_3 (99.997% trace metal bases, Thermo Scientific) and Cr_2O_3 (99.97%, chemPUR), in air at 1200 °C for 8 h after grinding at least for 30 minutes. An additional sample of ruby with < 1ppm activator fractions of Cr^{3+} was obtained by thoroughly grinding γ-Al_2O_3 for 20 minutes followed by heating in air at 1200°C for 8h for. The samples were identified as pure α-Al_2O_3 according to Rietveld refinement of the powder X-ray diffraction pattern (using the TOPAS 4.2 software) that was measured in a capillary in transmission mode (α-Al_2O_3: < 1 ppm Cr^{3+}) on a Stoe Stadi P instrument with a Cu$K_{\alpha 1}$-source (wavelength: 1.5406 Å, Ge(111) monochromator) (see Fig. S3a) and in reflection mode (α-$Al_{1.993}Cr_{0.007}O_3$) on a Malvern Panalytical instrument with a CuK_{α}-source (see Fig. S3b) in a 2θ range of 5 – 90° (see Tab. S1).

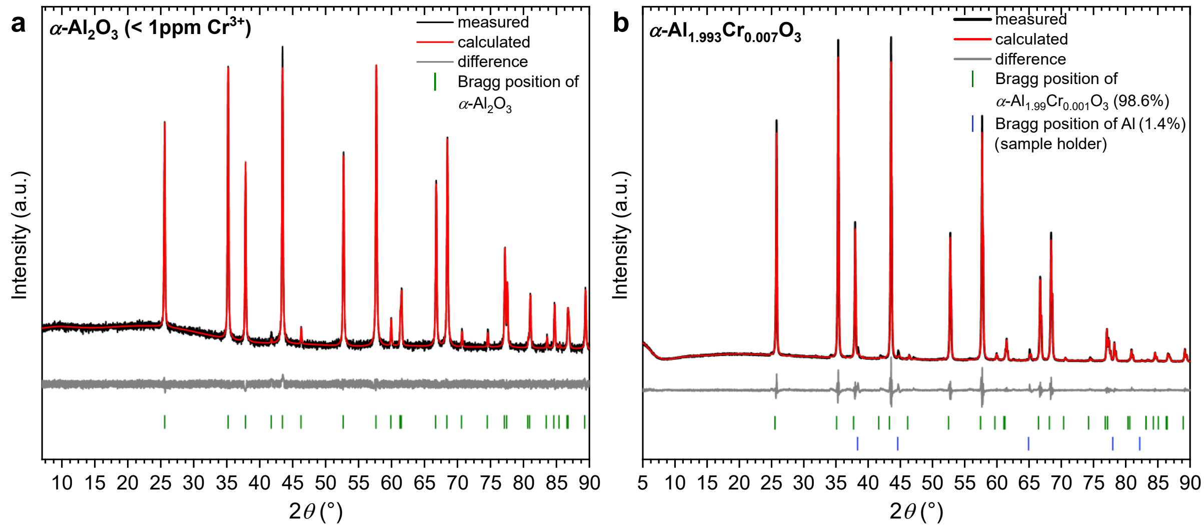

Figure S3. Rietveld refined X-ray powder diffraction patterns (Cu-$K_{\alpha 1}$) of the powdered sample of (a) α-Al_2O_3 (< 1ppm Cr^{3+}) and (b) α-$Al_{1.993}Cr_{0.007}O_3$ synthesized at 1200 °C by phase transition from γ-Al_2O_3 on metal basis (99.997%). The refinement was performed with the TOPAS 4.2 software[6].

Table S1. Structural data of $Al_{0.993}Cr_{0.007}B_4O_6N$, α-Al_2O_3 (< 1ppm Cr^{3+}) and α-$Al_{1.993}Cr_{0.007}O_3$ obtained from Rietveld refinement of the X-ray powder diffraction data (standard deviations in parentheses).

Empirical formula	$Al_{0.993}Cr_{0.007}B_4O_6N$	$Al_{0.993}Cr_{0.007}B_4O_6N$	α-Al_2O_3	α-$Al_{1.993}Cr_{0.007}O_3$
Space group	$P6_3mc$ (no. 186)	$P6_3mc$ (no. 186)	$R\bar{3}c$ (no. 167)	$R\bar{3}c$ (no. 167)
Crystal system	hexagonal	hexagonal	hexagonal	hexagonal
Molar mass, g/mol	180.235	180.235	101.961	101.961
Radiation; wavelength λ, pm	Mo$K_{\alpha 1}$; 70.93	Mo$K_{\alpha 1}$; 70.93	Cu$K_{\alpha 1}$; 154.05	Cu$K_{\alpha 1}$; 154.05<br>Cu$K_{\alpha 2}$; 154.44
a, pm	506.129(9)	503.679(8)	474.99(3)	476.44(6)
c, pm	827.96(3)	823.83(2)	1296.70(6)	1300.63(9)
V, Å^3	183.679(9)	180.998(7)	253.36(3)	255.69(1)
2θ range, °	2.0 – 60.5	2.0 – 60.5	7.00 – 89.99	5.00-90.00
2θ step width, °	0.015	0.015	0.01	0.0086
R_{exp}, %	2.22	2.95	6.40	3.85
R_{wp}, %	2.51	3.33	7.94	8.05
R_p, %	1.80	2.51	6.12	5.97
Goodness-of-fit	1.13	1.13	1.24	2.09
Calculated quantity ratio, wt%	53 $Al_{0.993}Cr_{0.007}B_4O_6N$<br>29 h-BN<br>18 c-BN	92 $Al_{0.993}Cr_{0.007}B_4O_6N$<br>6 H_3BO_3<br>2 h-BN	100 α-Al_2O_3<br>(< 1 ppm Cr^{3+})	99 α-$Al_{1.993}Cr_{0.007}O_3$<br>1 Al (sample holder)

Single-crystal $Al_{0.97}Cr_{0.03}B_4O_6N$. To exclude possible structural changes at low temperatures, a colorless single-crystal of $Al_{0.97}Cr_{0.03}B_4O_6N$ was measured at a temperature of 90(2) K with a Bruker D8 Quest diffractometer (Bruker Corporation, Billerica, Massachusetts, U.S.) equipped with a Photon III C14 pixel array detector. The programs SAINT (V8.40B)[9] and APEX4 (v2021.4.0)[10] were used for data collection and processing. A multi-scan absorption correction

was performed using SADABS (2016/2)[11]. For structure solution and parameter refinement, the software tools SHELXS (2008)[12] and SHELXL-2018/3 [13] integrated into the program Olex2-1.5 [14] were applied. $Al_{0.97}Cr_{0.03}B_4O_6N$ was solved and refined in the space group $P6_3mc$ (no. 186). All atoms could be refined anisotropically. Compared to the same crystal measured at 300(2) K by Widmann *et al.*[4], no phase transition or decomposition was observed at 90 K and both measurements clearly showed the same site symmetry. Details of the data collection can be found in the synoptical Tab. S2. CSD 2476833 ($Al_{0.97}Cr_{0.03}B_4O_6N$ at 90 K) contains the supplementary crystallographic data for this paper. The data can be obtained free of charge from The Cambridge Crystallographic Data Centre via www.ccdc.cam.ac.uk/data_request/cif.

Table S2: Single-crystal data and structure refinement of $Al_{0.97}Cr_{0.03}B_4O_6N$ at 90 K (middle column, present study) in comparison to reported data of the same crystal at 300 K by Widmann *et al.*[4] (right column). Standard deviations are specified in parentheses and refer to the last decimal place.

$Al_{0.97}Cr_{0.03}B_4O_6N$	90(2) K (present study)	300(2) K (Widmann *et al.*[4])
CSD number	2476833	2269930
Molar mass, g mol^{-1}	180.87	
Crystal system	hexagonal	
Space group	$P6_3mc$ (no. 186)	
Single-crystal diffractometer	Bruker D8 Quest	
Radiation / wavelength λ, pm	Mo-$K\alpha$ / 71.073	
a, pm	504.71(2)	505.06(2)
c, pm	823.89(4)	824.97(3)
V, nm^3	0.18175(2)	0.18225(2)
Formula units per cell Z	2	
Calculated density, g cm^{-3}	3.305	3.296
Crystal size, µm^3	80 × 40 × 20	
Temperature, K	90(2)	300(2)
Absorption coefficient, mm^{-1}	0.583	0.582
F(000), e	177	
θ range, °	4.66–40.30	4.66–37.05
Range in hkl	±9; ±9; +14/–15	±8; ±8; ±13
Refl. total / independent	7284 / 473	8135 / 394
R_{int} / R_σ	0.0453 / 0.0215	0.0283 / 0.0126

Refl. with $I > 2\,\sigma(I)$	462	386
Data / ref. parameters	473 / 29	394 / 29
Absorption correction	multiscan	
Final $R1$ / $wR2$ ($I > 2\,\sigma(I)$)	0.0207 / 0.0502	0.0172 / 0.0466
Final $R1$ / $wR2$ (all data)	0.0213 / 0.0504	0.0174 / 0.0467
Goodness-of-fit on F^2	1.180	1.242
Largest diff. peak / hole, e Å^{-3}	0.38 / −0.28	0.31 / −0.35

1.2. Photoluminescence spectroscopy

All temperature-dependent spectral data with $T \geq 77$ K were recorded on a photoluminescence spectrometer FLS1000 from Edinburgh Instruments with a continuous-wave 450 W Xe arc lamp as excitation source, blazed excitation (blaze: 400 nm) and emission (blaze: 500 nm) grating double monochromators (focal length 2 x 325 mm) in a Czerny-Turner configuration and a thermoelectrically cooled (-20 °C) photomultiplier tube PMT-980 (Hamamatsu Photonics). The samples were placed on a quartz plate into a Linkam THMS600 temperature cell (temperature range 78 K – 873 K) that was coupled with the sample chamber of the FLS1000 with optical fibre bundles. The temperature accuracy of the cell is ± 0.1 K. Both excitation and emission spectra were corrected with respect to the grating efficiency and the sensitivity of the detector, while the excitation spectra were additionally corrected with respect to the lamp intensity. All emission spectra were recorded between 78 K and 873 K in steps of $\Delta T = 25$ K steps. The temperature-dependent high-resolution emission spectra of the compounds $Al_{0.97}Cr_{0.03}B_4O_6N$ and α-$Al_{1.993}Cr_{0.007}O_3$ were obtained by exciting with the equipped 450 W Xe arc lamp. The time-resolved measurements for $Al_{0.97}Cr_{0.03}B_4O_6N$ was performed by exciting with a CNI laser diode (MDL375-III, λ_{em} = 375 nm) in pulsed mode with variable repetition rate between 0.1 Hz and 2 kHz, adjustable short pulse

width of 2 μs to 350 μs and a maximum incident beam power of 450 mW from the company Photontec Berlin. For α-Al_2O_3 (< 1 ppm Cr^{3+}), time-resolved measurements were acquired on an FS5 spectrofluorometer (Edinburgh Instruments) equipped with a 150 W Xe μs-lamp with variable repetition rate (temporal pulse width *ca*. 2 μs), single-grating monochromators, (focal length 225 mm) and a thermoelectrically cooled photomultiplier tube R928P. The sample was also placed into a Linkam THMS600 temperature cell coupled to the chamber of the FS5 with optical fibres. Single-photon multi-channel scaling was used as the detection mode for all time-resolved measurements.

Excitation for spectral data below liquid nitrogen temperature was performed with an external pulsed wavelength-tunable Opotek Opolette HE 355 II (Carlsbad, CA, USA) optical parametric oscillator (OPO) pumped by a frequency-tripled Nd:YAG laser at a repetition rate of 20 Hz and a temporal pulse width of approximately 6 ns. The photoluminescence spectra below 77 K were recorded on an Edinburgh Instrument FLS920 spectrofluorometer (Livingston, UK) equipped with a 0.25 m single Littrow-configuration grating monochromator blazed at 300 nm and a Hamamatsu R928P (Shizuoka, Japan) photomultiplier tube (PMT) for photon detection. All emission spectra were corrected for grating efficiency and detector sensitivity. Temperature-dependent measurements below room temperature were performed with an Oxford Instruments liquid He flow cryostat (Oxford, UK) and an external temperature control unit, which measured the temperature by means of a thermocouple in direct contact with the powder sample holder. Photoluminescence decay curves were acquired by pulsed excitation with the pump laser of the OPO (λ_{ex} = 355 nm) and detection of the time-resolved signal with a single-photon multichannel scaler (MCS) attached to a Hamamatsu H7422 PMT (Shizuoka, Japan) for minimized background.

The Tanabe-Sugano diagram based on the estimated Racah and ligand field parameters from the experimental high-resolution excitation spectra were generated with the program TanabeSugano[15].

2. Results

2.1. Photoluminescence spectroscopy at 20 K

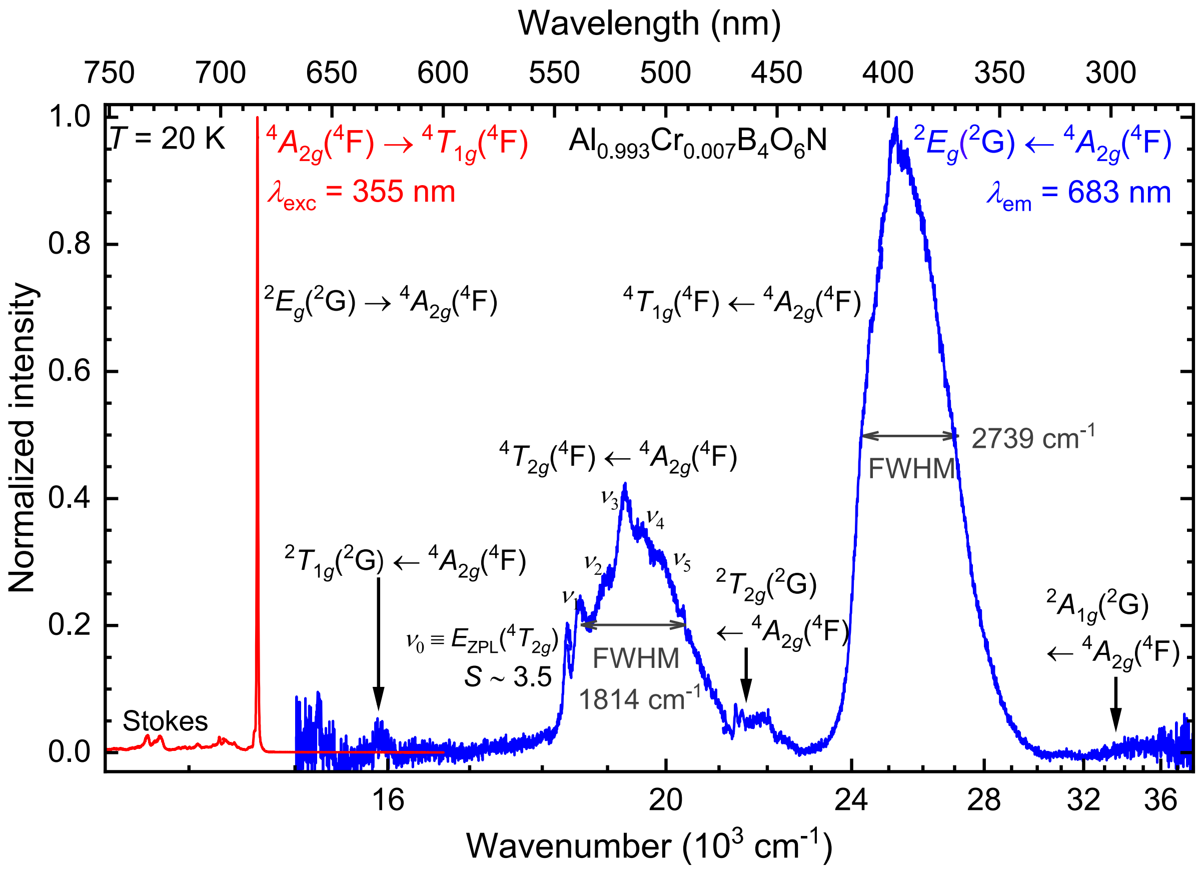

Figure S4. High-resolution excitation (blue, λ_{em} = 683 nm) and emission (red, λ_{exc} = 355 nm) spectra of $Al_{0.993}Cr_{0.007}B_4O_6N$ at T = 20 K. The energies of the assigned electronic transitions are compiled in Table S3. The zero-phonon line (ZPL) and vibronic progression of the $^4T_{2g}$ (^{4}F) ← $^4A_{2g}$ (^{4}F) excitation transition are indicated from the transition 0-0 to 0-5 (see Table S4). Intensities were converted to the wavenumber scale with Jacobian correction. S denotes the Huang-Rhys-Pekar parameter, which can be estimated by counting the number of vibronic lines assuming a Pekarian line shape.

Table S3. Optical transitions from the excitation and emission spectrum of $Al_{0.993}Cr_{0.007}B_4O_6N$ at T = 20 K. Additional values obtained at T = 78 K are listed in gray color.

Assignment		Wavenumber / cm^{-1}	Wavelength / nm
^2E_g (^{2}G) → $^4A_{2g}$ (^{4}F)		14641	682.9
^2E_g (^{2}G) ← $^4A_{2g}$ (^{4}F)		14665 (78 K)	681.9 (78 K)
$^2T_{1g}$ (^{2}G)	Splits into	15255	655.5
	$\Gamma_6^+ \oplus \Gamma_8^+$	15267	655.0
		- (15333)	652.2
$^4T_{2g}$ (^{4}F)		18369	555.0
$^2T_{2g}$ (^{2}G)	Splits into	21400 (21336)	467.2 (467.2)
	$\Gamma_7^+ \oplus \Gamma_8^+$	(21436)	465.7
		21533 (21480)	464.5 (464.4)
$^4T_{1g}$ (^{4}F) (maximum)		25374	391.8
$^4T_{1g}$ (^{4}F) (zero-phonon line)		~23375 (estimated)	~420.3 (estimated)
ΔE_{21} (20 K)		614	-
ΔE_{31} (20 K)		3728	-

Table S4. Vibronic fine structure of the excitation transition $^4T_{2g}$ (^{4}F) ← $^4A_{2g}$ (^{4}F) for $Al_{0.993}Cr_{0.007}B_4O_6N$ at T = 20 K and T = 78 K from Widmann *et al.*[4].

ν($^4T_{2g}$ (^{4}F))	Wavenumber / cm^{-1}		$\Delta\nu$ / cm^{-1}	$\Delta\nu$ / cm^{-1}
	T = 20 K	T = 78 K	T = 20 K	T = 78 K
0-0	18369	18355	331	322
0-1	18619	18605	250	250
0-2	18993	18990	374	385
0-3	19331	19314	338	324
0-4	19627	19619	296	305
0-5	19956	19942	329	323

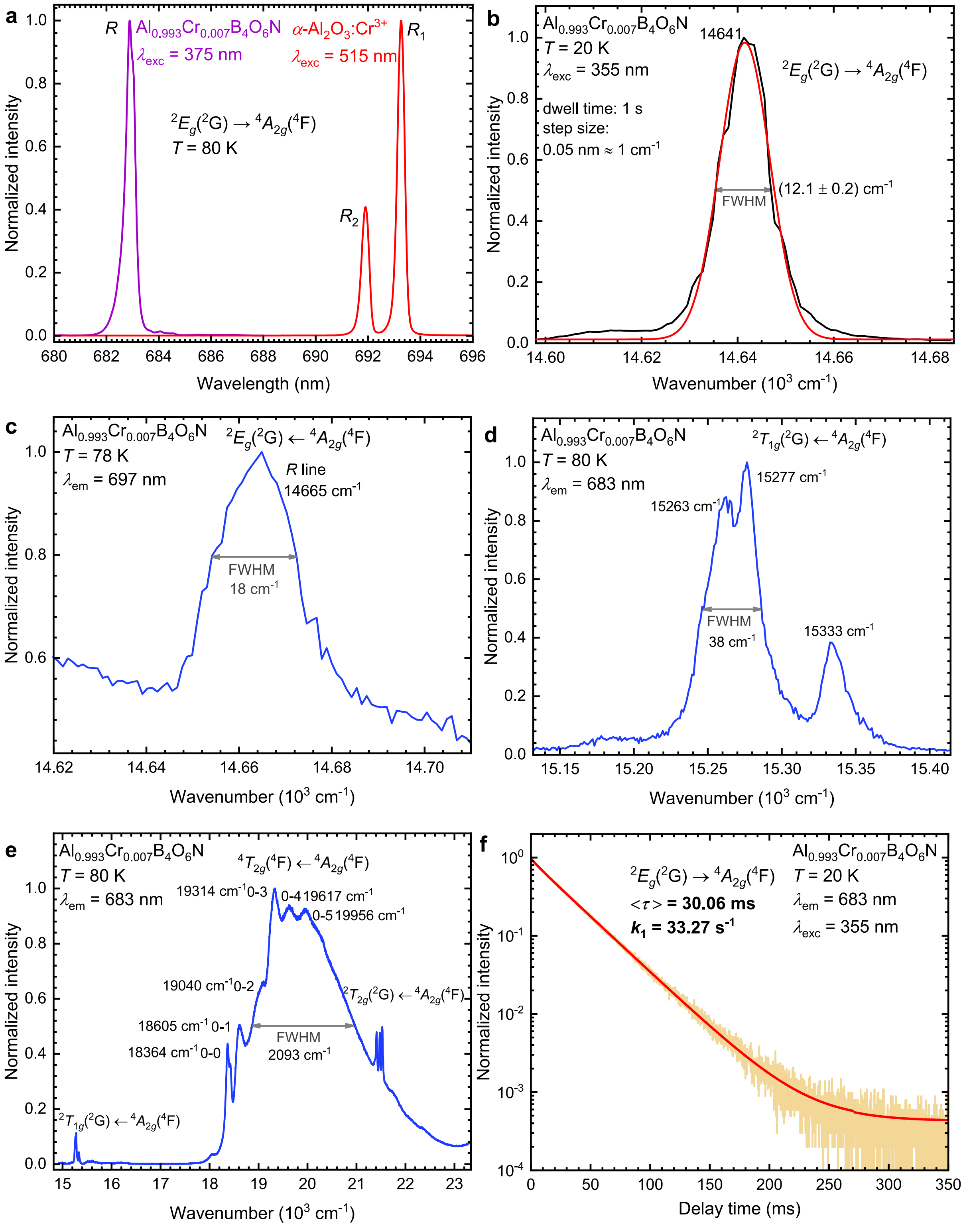

Figure S5. *Top panel*: High-resolution a emission spectra of the *R* lines of $Al_{0.993}Cr_{0.007}B_4O_6N$ and α-$Al_2O_3:Cr^{3+}$ at T = 80 K and b of $Al_{0.993}Cr_{0.007}B_4O_6N$ at T = 20 K. The red line is a Gaussian fit to the line. *Middle panel*: High-resolution excitation spectra at 80 K depicting c the $^2E_g(^2G) \leftarrow {}^4A_{2g}(^4F)$ and d the $^2T_{1g}(^2G) \leftarrow {}^4A_{2g}(^4F)$ transition in $Al_{0.993}Cr_{0.007}B_4O_6N$. *Bottom panel*: e High-

resolution excitation spectrum depicting the ${}^4T_{2g}({}^4\mathrm{F}) \leftarrow {}^4A_{2g}({}^4\mathrm{F})$-based transition with pronounced vibronic fine structure. The detection of two different vibrational modes is a consequence of the excited-state $T_{2(g)} \otimes e$-type Jahn-Teller coupling[16], which also gives rise to the larger FWHM= 2093 cm^{-1} for that excitation band in $Al_{0.993}Cr_{0.007}B_4O_6N$ compared to α-$Al_2O_3:Cr^{3+}$ (see Fig. S6). f Decay trace of the ${}^2E_g({}^2\mathrm{G}) \rightarrow {}^4A_{2g}({}^4\mathrm{F})$-based emission. The solid line is a double exponential fit to the data.

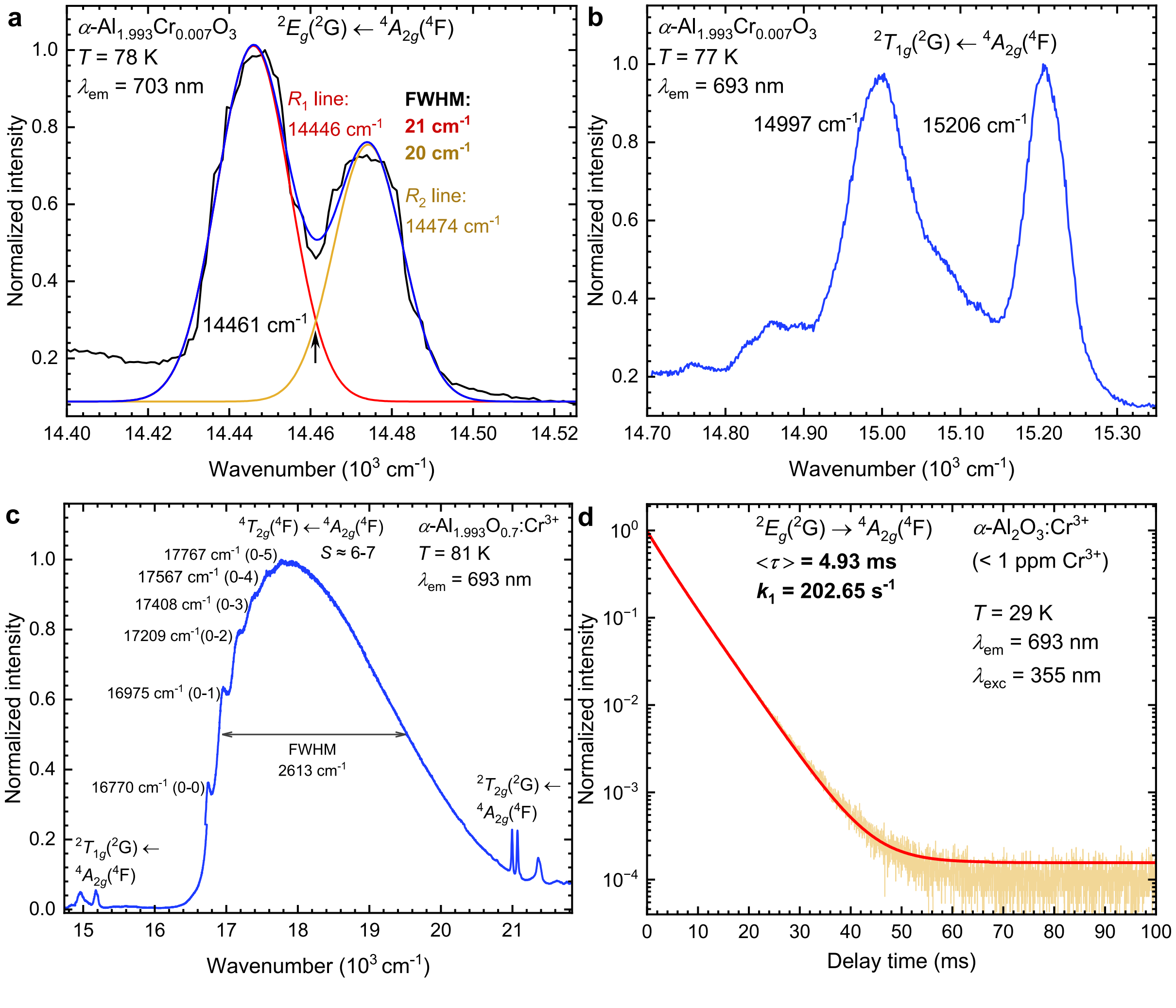

Figure S6. High-resolution excitation spectra in α-$Al_{1.993}Cr_{0.007}O_3$ (ruby) at around T = 80 K depicting a the ${}^2E_g({}^2\mathrm{G}) \leftarrow {}^4A_{2g}({}^4\mathrm{F})$, b the ${}^2T_{1g}({}^2\mathrm{G}) \leftarrow {}^4A_{2g}({}^4\mathrm{F})$ and c the ${}^4T_{2g}({}^4\mathrm{F}) \leftarrow {}^4A_{2g}({}^4\mathrm{F})$ transition with pronounced vibronic fine structure. d Decay trace of the ${}^2E_g({}^2\mathrm{G}) \rightarrow {}^4A_{2g}({}^4\mathrm{F})$-based emission at 29 K. The solid line is a double exponential fit to the data.

Table S5. Optical transitions from the excitation and emission spectrum of α-$Al_{1.993}Cr_{0.007}O_3$ at T = 80 K.

Assignment		Wavenumber / cm^{-1}	Wavelength / nm
^2E_g (^{2}G) → $^4A_{2g}$ (^{4}F)		14424	693.3
		14453	691.2
^2E_g (^{2}G) ← $^4A_{2g}$ (^{4}F)		14446	692.2
		14474	690.9
$^2T_{1g}$ (^{2}G)	Splits into	14997	666.6
	$\Gamma_6^+ \oplus \Gamma_8^+$	15206	657.7
$^4T_{2g}$ (^{4}F)		16768	596.4
$^2T_{2g}$ (^{2}G)	Splits into	20995	476.3
	$\Gamma_7^+ \oplus \Gamma_8^+$	21068	474.7
		21359	468.1
$^4T_{1g}$ (^{4}F) (maximum)		23375	400.4
$^4T_{1g}$ (^{4}F) (zero-phonon-line)		~23640 (estimated)	~430.4 (estimated)
ΔE_{21}		544 (minimum distance)	-
ΔE_{31}		2296 (minimum distance)	-

2.2. Ratiometric (quasi-)Boltzmann thermometry in α-$Al_{1.993}Cr_{0.007}O_3$ (ruby)

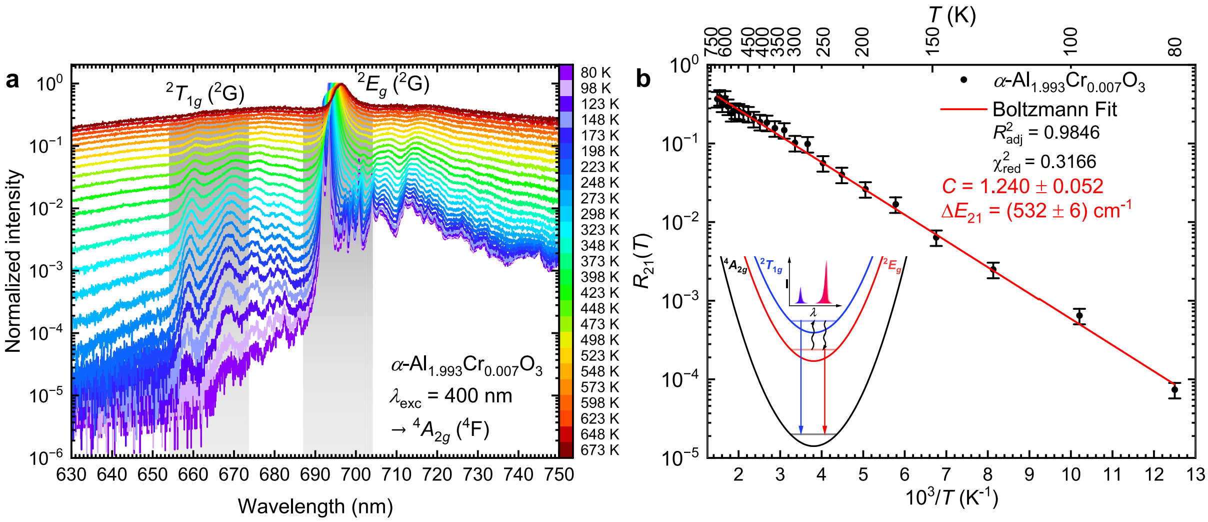

Figure S7. Boltzmann-type luminescence thermometry with Cr^{3+} in α-$Al_{1.993}Cr_{0.007}O_3$ exploiting the narrow-line emission due to the $^2T_{1g}(^2G) \rightarrow {}^4A_{2g}(^4F)$- and $^2E_g(^2G) \rightarrow {}^4A_{2g}(^4F)$-based emission lines. a Normalized temperature-dependent normalized emission spectra in semi-logarithmic scale. The integration ranges are shifted with the temperature and are shaded in gray color, respectively. b Plot of the luminescence intensity ratio $R_{21}(T)$ of the integrated intensities of the $^2T_{1g}(^2G) \rightarrow {}^4A_{2g}(^4F)$ and $^2E_g(^2G) \rightarrow {}^4A_{2g}(^4F)$-based emission lines of α-$Al_{1.993}Cr_{0.007}O_3$ between 650 to 675 nm and 685 to 700 nm, respectively. The red line is the least-squares fit to Eq. 1 in the main manuscript. The inset shows illustratively the configurational coordinate diagram for Boltzmann-type thermometers based on photoluminescence emission of spectrally distinguishable two narrow-lines.

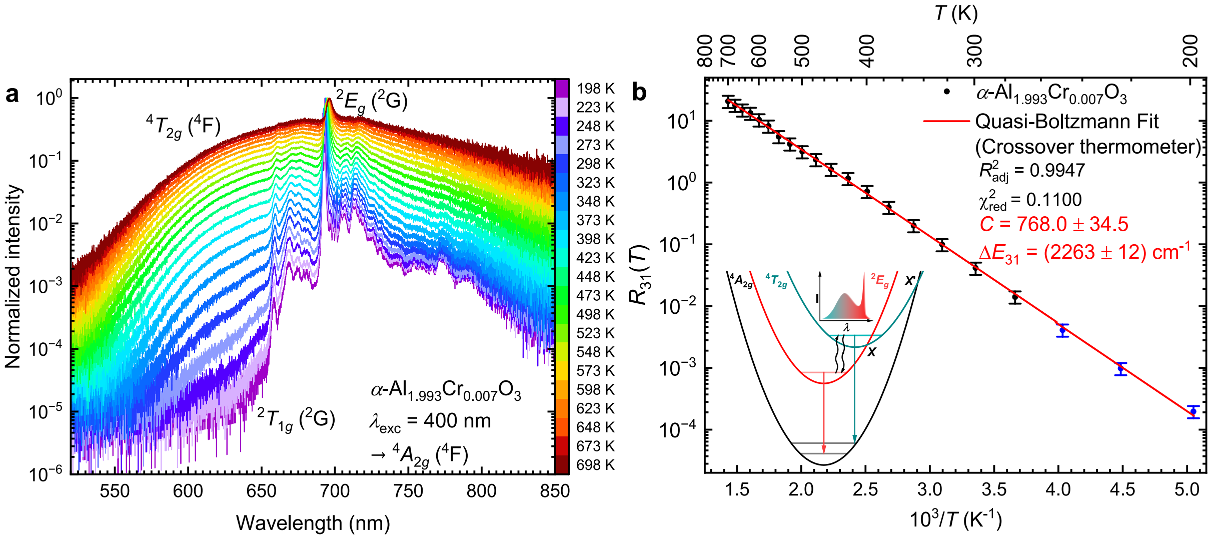

Figure S8. Quasi-Boltzmann-type luminescence thermometry with Cr^{3+} in α-$Al_{1.993}Cr_{0.007}O_3$ exploiting the narrow-line emission due to the $^4T_{2g}(^4F) \rightarrow {}^4A_{2g}(^4F)$- and $^2E_g(^2G) \rightarrow {}^4A_{2g}(^4F)$-based emission lines. a Normalized temperature-dependent emission spectra in semi-logarithmic scale. b Plot of the luminescence intensity ratio $R_{31}(T)$ of the integrated intensities of the $^4T_{2g}(^4F) \rightarrow {}^4A_{2g}(^4F)$-based broad-band and $^2E_g(^2G) \rightarrow {}^4A_{2g}(^4F)$-based narrow emission line between 450 to 850 nm and 685 to 700 nm (slightly varied dependent on the position of the zero-phonon line), respectively. The red line is the least-squares fit to Eq. 6 in the main manuscript with $A \rightarrow 0$. The blue LIR points are obtained by triangulation of the integrals underneath the $^4T_{2g}(^4F) \rightarrow {}^4A_{2g}(^4F)$-related broad band. The inset shows illustratively the configurational coordinate diagram with the involved crossover points X and X' in Crossover thermometers based on a narrow-line and a broad-band emission, respectively.

2.2.1. Temperature-dependent time-resolved luminescence in $Al_{0.993}Cr_{0.007}B_4O_6N$ and α-Al_2O_3:Cr^{3+} (< 1ppm Cr^{3+})

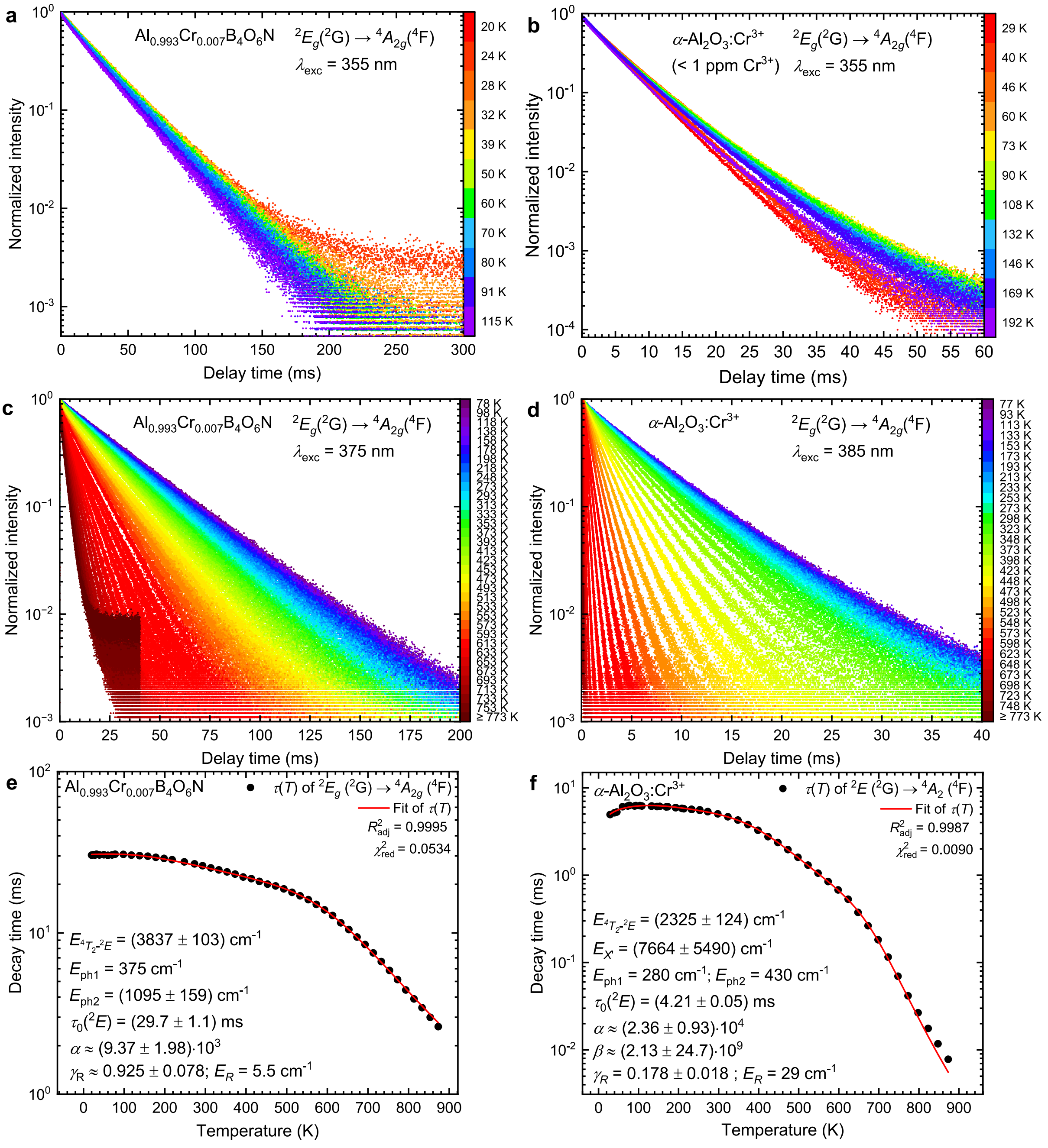

Figure S9. Temperature-dependent decay times of the $^2E_{(g)}(^2G)$ state in $Al_{0.993}Cr_{0.007}B_4O_6N$ (*left column*) and α-Al_2O_3 (< 1ppm Cr^{3+}) (*right column*) in semi-logarithmic scale corresponding for the range between a, b T = 20 K and T = 273 K with a liquid-flow He cryostat as well as c, d T = 78 K and T = 873 K in a Linkam temperature stage. e, f Temperature-dependent evolution of the derived decay times. The red line depicts a least-squares fit according to Eq. S1. Note the

initial increase in decay time in the case of α-Al_2O_3 (< 1ppm Cr^{3+}) between 20 K and 50 K, which is related to slightly different radiative decay rates from the two Kramers' doublets $2\bar{\mathbf{A}}$ and $\bar{\mathbf{E}}$ as originally explained by Nelson and Sturge[17].

In Figure 12 the temperature-dependent decay times of the *R* line emission are fitted to the model according to Eq. S1 as predicted by us[4,18] for both compounds (see bottom panels):

$$\tau(T) = \tau_0(^2E)\frac{1+\exp\left(-\frac{\Delta E_\mathrm{R}}{k_\mathrm{B}T}\right)+6\cdot\exp\left(-\frac{\Delta E_{^4T_2-^2E}}{k_\mathrm{B}T}\right)}{\coth\left(\frac{\hbar\omega_1}{2k_\mathrm{B}T}\right)+\gamma_\mathrm{R}\coth\left(\frac{\hbar\omega_2}{2k_\mathrm{B}T}\right)\exp\left(-\frac{\Delta E_\mathrm{R}}{k_\mathrm{B}T}\right)+\alpha\cdot\exp\left(-\frac{\Delta E_{^4T_2-^2E}}{k_\mathrm{B}T}\right)+\beta\cdot\exp\left(-\frac{\Delta E_{\mathrm{X}'}+\Delta E_{^4T_2-^2E}}{k_\mathrm{B}T}\right)} \quad \text{(S1)}$$

with $\alpha = \frac{\tau_0(^2E)}{\tau_0(^4T_2)} = \frac{k_\mathrm{r}(^4T_2)}{k_\mathrm{r}(^2E)}$ and $\beta = \frac{\tau_0(^2E)}{\tau_{\mathrm{X}'}} = \frac{k_{\mathrm{X}'}}{k_\mathrm{r}(^2E)}$, k_B the Boltzmann constant, $\hbar\omega_1$ and $\hbar\omega_2$ are the energies of the two effective phonon modes with most dominant coupling contribution (in Figs. S12e and f denoted as E_{ph1} and E_{ph2}), γ_R is the intensity ratio of the two respective phonon sidebands ($\gamma_\mathrm{R} \approx 0.65$)[18,19], ΔE_R = 29.1 cm^{-1} is the splitting between the two Kramers' doublets $2\bar{\mathrm{A}}$ ($\Gamma_{5,6}$) and $\bar{\mathrm{E}}$ (Γ_4), $\Delta E_{^4T_2-^2E}$ the mutual adiabatic energy gap between the $^2E_{(g)}(^2\mathrm{G})$ and $^4T_{2(g)}(^4\mathrm{F})$ states, τ_0 the intrinsic radiative lifetime of a respective state and $\Delta E_{X'}$ the crossover barrier for thermal quenching. The estimated quenching temperature $T_{1/2}$ for $Al_{0.993}Cr_{0.007}B_4O_6N$ is $T_{1/2} \approx 570$ K, while the one for α-Al_2O_3 (< 1ppm Cr^{3+}) is $T_{1/2} \approx 463$ K.

2.2.2. Relative sensitivities $S_r(T)$ and temperature uncertainties σ_T/T of $Al_{0.993}Cr_{0.007}B_4O_6N$ and α-$Al_{1.993}Cr_{0.007}O_3$

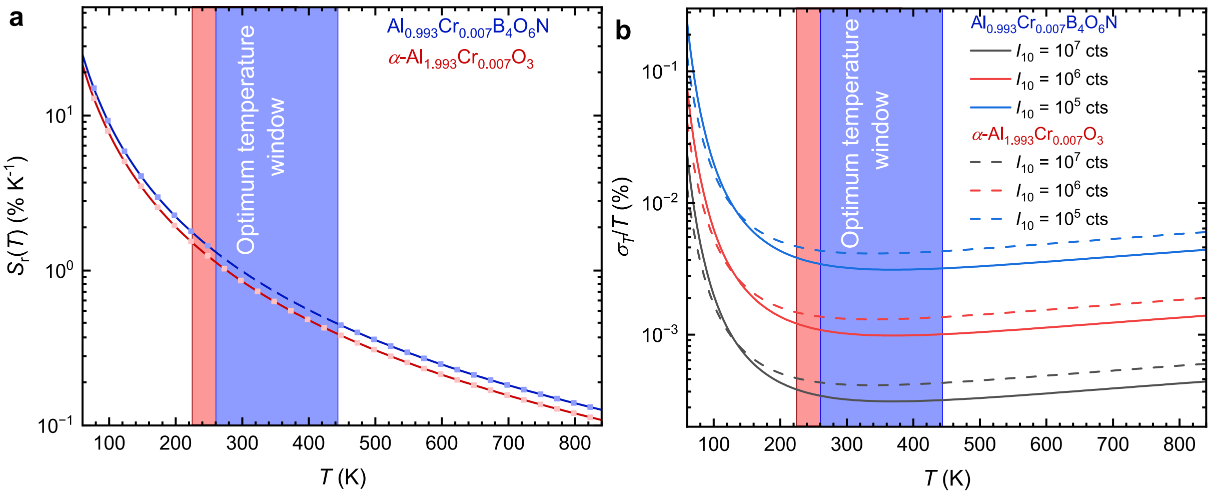

Figure S10. Performance analysis of the ratiometric luminescence thermometry concept with the ${}^2T_{1g}({}^2G) \rightarrow {}^4A_{2g}({}^4F)$- and ${}^2E_g({}^2G) \rightarrow {}^4A_{2g}({}^4F)$-based narrow line transitions of Cr^{3+}. a Relative sensitivity $S_r(T)$ of the ${}^2T_{1g}({}^2G)$-${}^2E_g({}^2G)$ gap in $Al_{0.993}Cr_{0.007}B_4O_6N$ (blue) and α-$Al_{1.993}Cr_{0.007}O_3$ (red). b Minimum statistical relative temperature readout uncertainty σ_T/T for different integrated photon counts I_{10} of the lower energetic ${}^2E_g({}^2G) \rightarrow {}^4A_{2g}({}^4F)$-based emission. The light-colored areas refer to the estimated optimum temperature range by Eq. 2 for $Al_{0.993}Cr_{0.007}B_4O_6N$ (blue) and α-$Al_{1.993}Cr_{0.007}O_3$ (red), respectively.

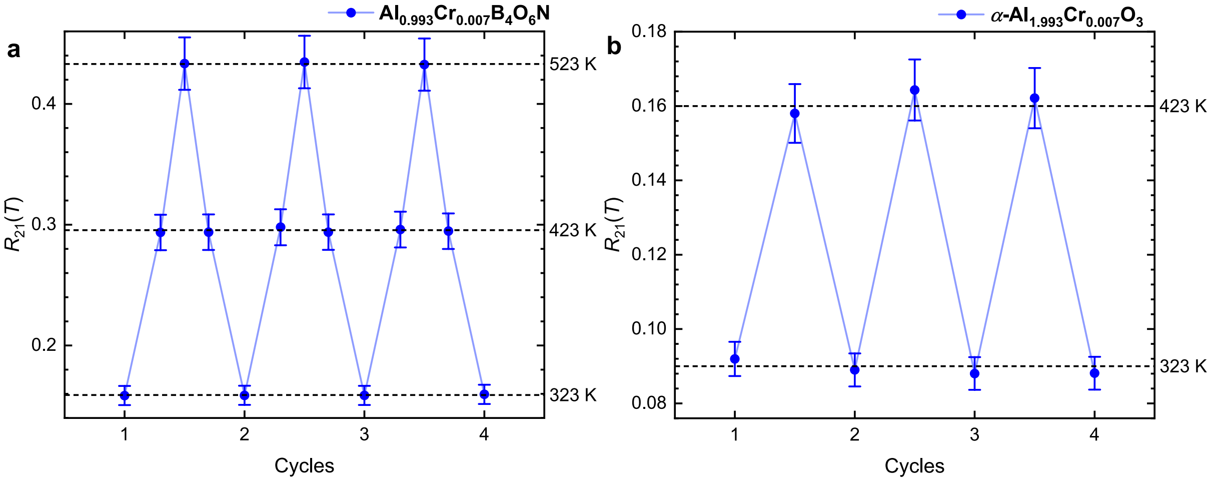

Figure S11. Cycle measurements for the Boltzmann thermometer $R_{21}(T)$ a $Al_{0.993}Cr_{0.007}B_4O_6N$ at temperatures 323 K, 423 K and 523 K and b α-$Al_{1.993}Cr_{0.007}O_3$ at temperatures 323 K and 423 K with at least 3 cycles. The errors were estimated assuming Poissonian photon counting statistics of the photomultiplier.

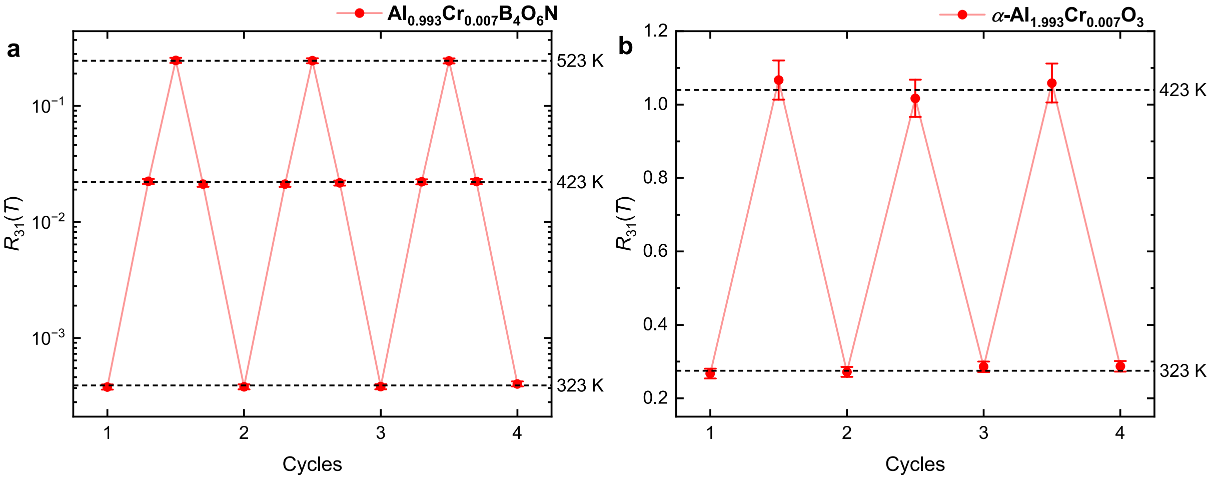

Figure S12. Cycle measurements for the Boltzmann thermometer $R_{31}(T)$ a $Al_{0.993}Cr_{0.007}B_4O_6N$ at temperatures 323 K, 423 K and 523 K and b α-$Al_{1.993}Cr_{0.007}O_3$ at temperatures 323 K and 423 K with at least 3 cycles. The errors were estimated assuming Poissonian photon counting statistics of the photomultiplier.

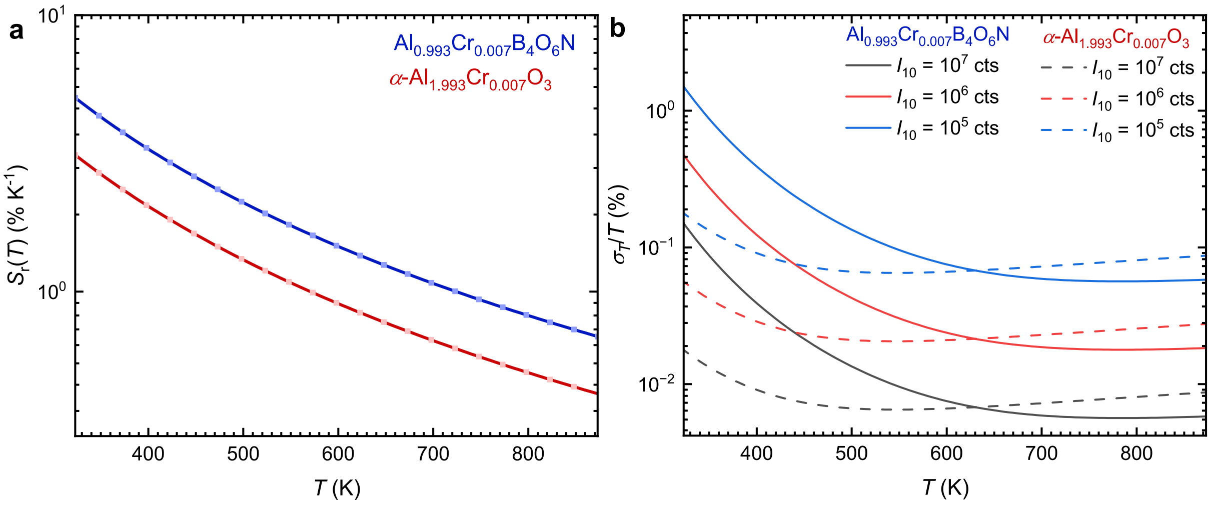

Figure S13. Performance analysis of the ratiometric luminescence thermometry concept with the $^4T_{2g}(^4F) \rightarrow {}^4A_{2g}(^4F)$ band and $^2E_g(^2G) \rightarrow {}^4A_{2g}(^4F)$ narrow line transitions of Cr^{3+}. a Relative sensitivity $S_r(T)$ of the $^4T_{2g}(^4F)$-$^2E_g(^2G)$ gap in $Al_{0.993}Cr_{0.007}B_4O_6N$ (blue) and α-$Al_{1.993}Cr_{0.007}O_3$ (red). b Minimum statistical relative temperature readout uncertainty σ_T/T for different integrated photon counts I_{10} of the lower energetic $^2E_g(^2G) \rightarrow {}^4A_{2g}(^4F)$-based emission.

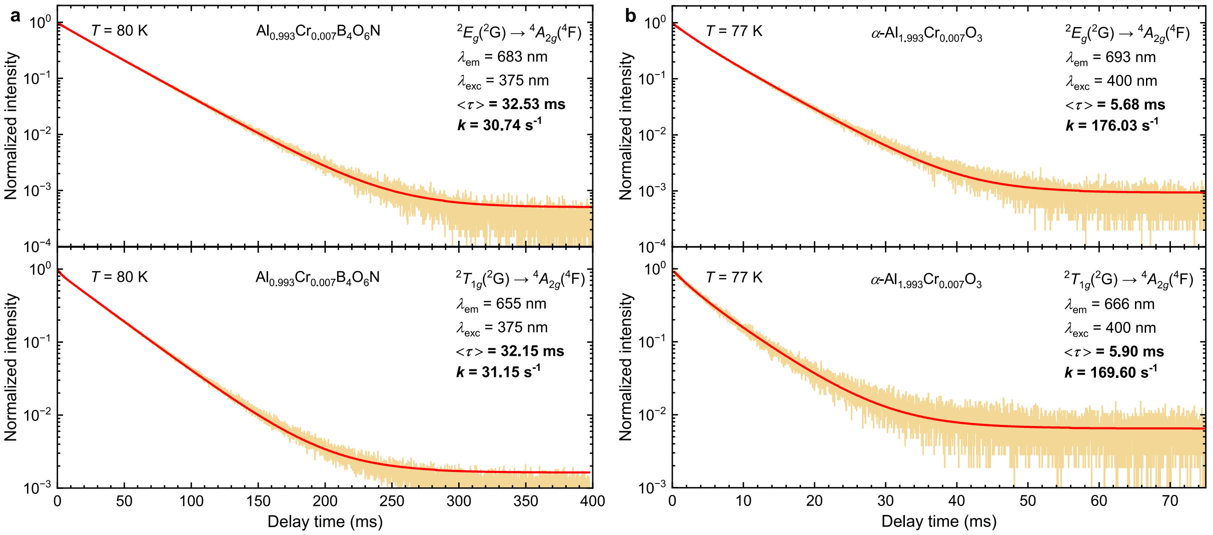

Figure S14. Decay traces of the $^2E_g(^2G) \rightarrow {}^4A_{2g}(^4F)$- (*top*) and the $^2T_{1g}(^2G) \rightarrow {}^4A_{2g}(^4F)$-based (*bottom*) emission in a $Al_{0.993}Cr_{0.007}B_4O_6N$ at T = 80 K and b α-$Al_{1.993}Cr_{0.007}O_3$ at 77 K demonstrating thermalization between the two excited states.

2.3. Broad-band $^4T_{2(g)}$ (^{4}F) → $^4A_{2g}$ (^{4}F)-based emission in $Al_{0.993}Cr_{0.007}B_4O_6N$ and α-$Al_{1.993}Cr_{0.007}O_3$

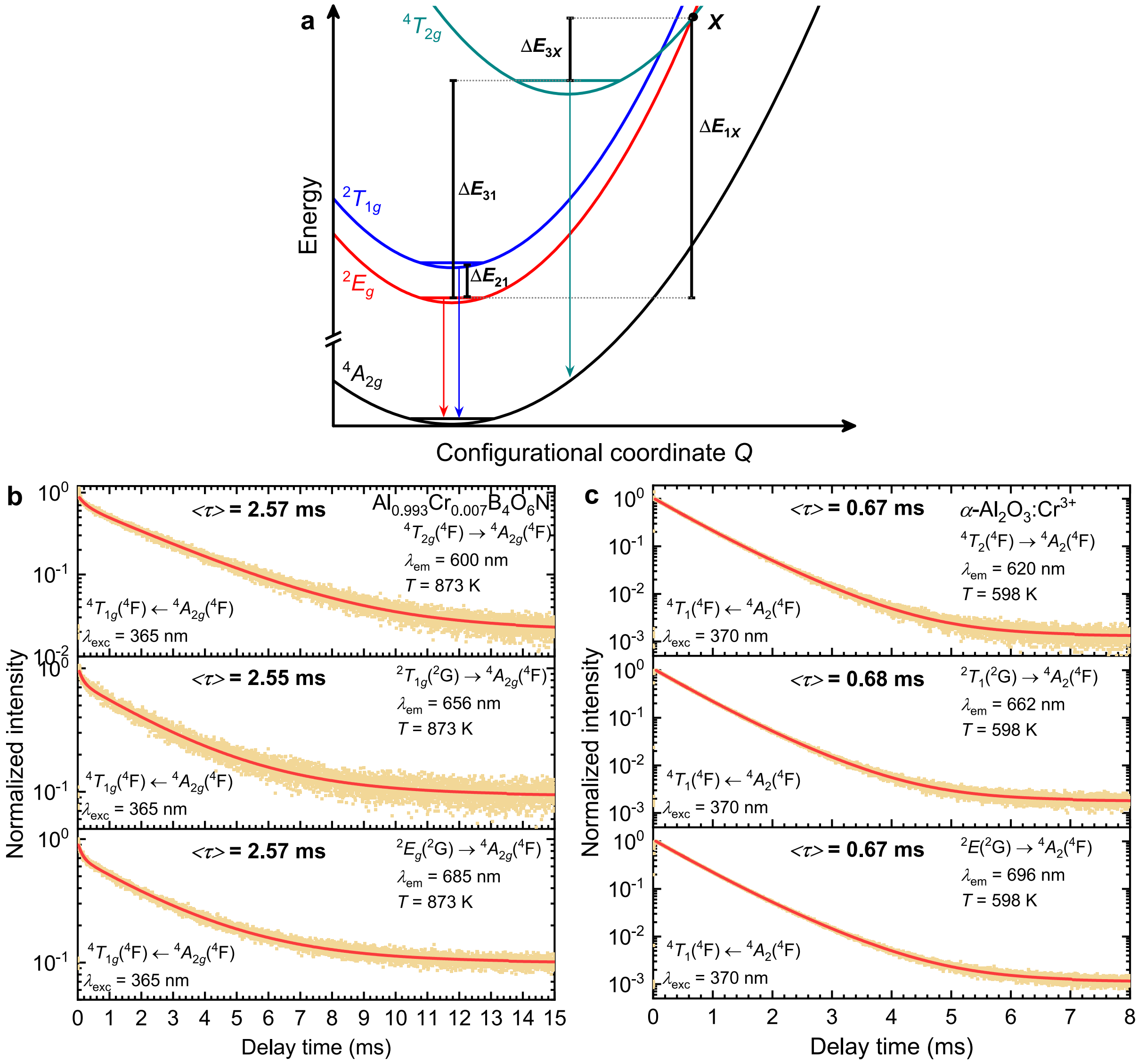

Figure S15. Nonradiative crossover processes in $Al_{0.993}Cr_{0.007}B_4O_6N$ and α-$Al_2O_3:Cr^{3+}$ (< 1ppm Cr^{3+}). a Configurational coordinate diagram depicting the potential energy curves for the thermometrically relevant emissive states of $Al_{0.993}Cr_{0.007}B_4O_6N$ and α-$Al_2O_3:Cr^{3+}$ (< 1ppm Cr^{3+}). b Decay traces of the $^4T_{2(g)}$ (^{4}F) → $^4A_{2g}$ (^{4}F)- (*top*), ^2E_g (^{2}G) → $^4A_{2g}$ (^{4}F)- (*medium*) and $^2T_{1g}$ (^{2}G) → $^4A_{2g}$ (^{4}F)-based (*bottom*) emission in $Al_{0.993}Cr_{0.007}B_4O_6N$ at T = 873 K demonstrating thermalization of the three states at that temperature. The short decay component is not

related to Cr^{3+} and probably due to unintentional defects. c Same decay traces for α-Al_2O_3:Cr^{3+} (< 1ppm Cr^{3+}) at T = 598 K. The lower temperature was necessary to avoid excessive thermal quenching.

The radiative decay time of the ${}^4T_{2g}({}^4F)$ state can be also estimated from Herzberg's perturbative spin-orbit mixing model[20-22]

$$d = \pm\sqrt{\frac{1}{2}\left(1 - \frac{\Delta E}{\sqrt{4\cdot|V_{SO}|^2 + \Delta E^2}}\right)} \tag{S2}$$

$$k_\mathrm{r}\left({}^4T_{2g}\right) = \frac{1}{\tau_\mathrm{r}\left({}^4T_{2g}\right)} = \frac{1}{\tau'\left({}^4T_{2g}\right)\cdot d^2} \tag{S3}$$

where V_{SO} is the spin-orbit coupling matrix element that couples the ${}^2E_g({}^2G)$ state with the ${}^4T_{2g}({}^4F)$ state, ΔE is the mutual (adiabatic 0–0) energy difference between these two states, and $\tau'\left({}^4T_{2g}\right)$ is the pure (unperturbed and non-observable) decay time of the ${}^4T_{2g}({}^4F)$ state.

In addition, the radiative decay time of the ${}^4T_{2g}({}^4F)$ state is retrieved by the temperature-dependent mean radiative rate $\langle k(T)\rangle$ by the radiative rate k_{1r} contribution and the resulting energy gap ΔE_{31} from the fitting results in Fig. 4 of the main article and Fig. S13.

$$\langle k(T)\rangle = \frac{k_{1r} + 3\,k_{3r}\exp\left(-\frac{\Delta E_{31}}{k_B T}\right)}{1 + 3\exp\left(-\frac{\Delta E_{31}}{k_B T}\right)} \tag{S4}$$

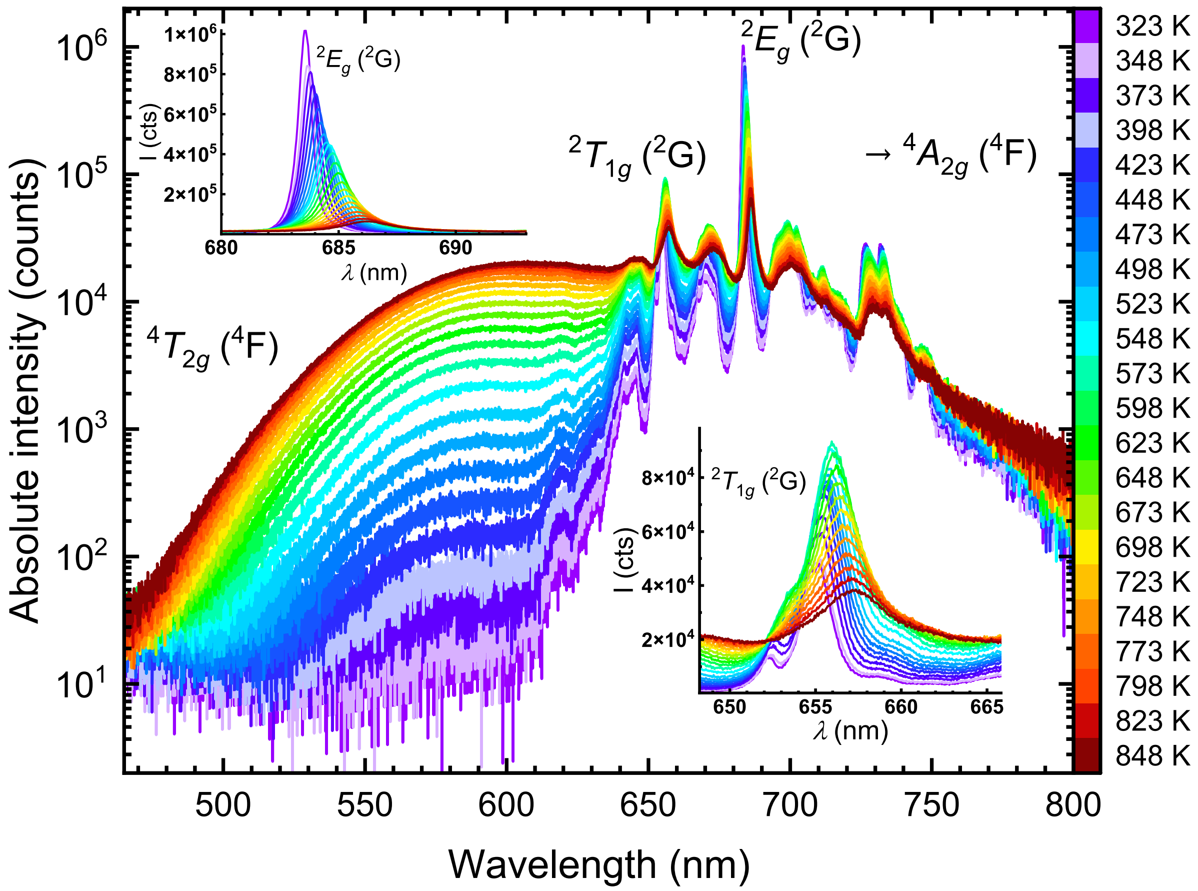

Figure S16. Temperature-dependent absolute emission spectra of the $Al_{0.993}Cr_{0.007}B_4O_6N$ excited at l_{exc} = 395 nm between 323 K and 848 K in a semi-logarithmic scale. The insets are representing the magnified spectral part for the $^2T_{1g}$ (^{2}G) ⟶ $^4A_{2g}$ (^{4}F)-based narrow-line emission (*right bottom*) and ^2E_g (^{2}G) ⟶ $^4A_{2g}$ (^{4}F)-based narrow-line emission (*left top*).

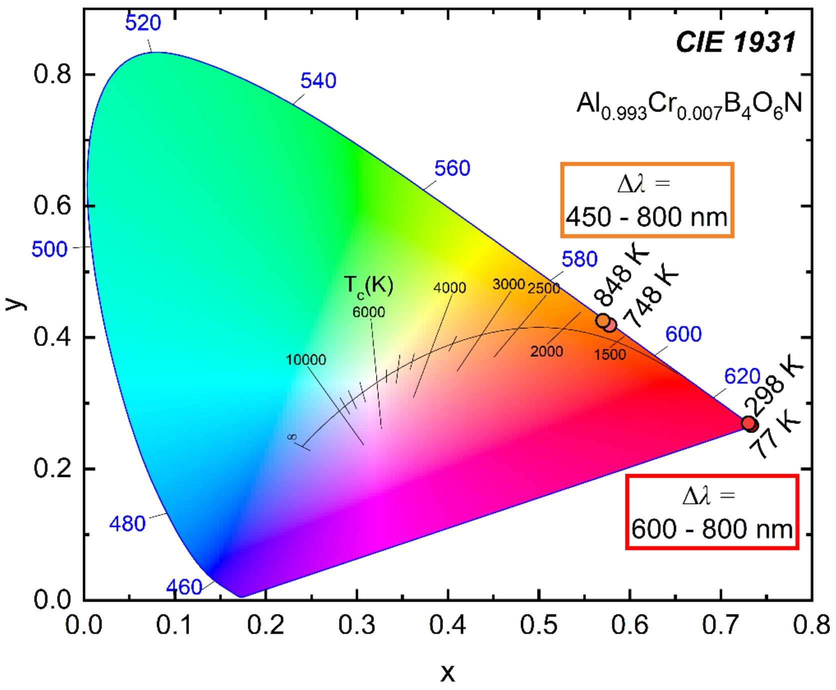

Figure S17. *CIE 1931* diagram depicting the color coordinates for the spectra of $Al_{0.993}Cr_{0.007}B_4O_6N$ at temperatures of 77 K, 298 K (red circles) and 748 K, 848 K (orange circles) with the respective integration ranges $\Delta\lambda$.

3. References

1. Huppertz, H. Multianvil high-pressure / high-temperature synthesis in solid state chemistry. *Z. Kristallogr. Cryst. Mater.* 219, 330-338, https://doi.org/10.1524/zkri.219.6.330.34633 (2004).
2. Walker, D. Lubrication, gasketing, and precision in multianvil experiments. *Am. Mineral.* 76, 1092-1100, (1991).
3. Walker, D., Carpenter, M. A. & Hitch, C. M. Some simplifications to multianvil devices for high pressure experiments. *Am. Mineral.* 75, 1020-1028, (1990).
4. Widmann, I. et al. Real competitors to ruby: the triel oxonitridoborates AlB_4O_6N, $Al_{0.97}Cr_{0.03}B_4O_6N$, and $Al_{0.83}Cr_{0.17}B_4O_6N$. *Adv. Funct. Mater.* 34, 2400054, https://doi.org/10.1002/adfm.202400054 (2024).
5. Rietveld, H. M. A profile refinement method for nuclear and magnetic structures. *J. Appl. Crystallogr.* 2, 65-71, https://doi.org/10.1107/s0021889869006558 (1969).
6. Topas 4.2 v. 4.2 (Bruker Analytical X-ray Instruments Inc., Madison, Winsconsin, USA, 2009).
7. Li, M.-R. et al. $Ba_3(Cr_{0.97(1)}Te_{0.03(1)})_2TeO_9$: in Search of Jahn-Teller Distorted Cr(II) Oxide. *Inorg. Chem.* 55, 10135–10142, https://doi.org/10.1021/acs.inorgchem.6b01047 (2016).
8. Will, G., Kirfel, A. & Josten, B. Charge density and chemical bonding in cubic boron nitride. *J. Alloys Compd.* 117, 61-71, https://doi.org/10.1016/0022-5088(86)90012-3 (1986).
9. SAINT (Bruker AXS Inc., Madison, Wisconsin (USA), 2021).
10. APEX4 (Bruker AXS Inc., Madison, Wisconsin (USA), 2021).
11. SADABS (Bruker AXS Inc., Madison, Wisconsin (USA), 2016).
12. Sheldrick, G. M. A short history of SHELX. *Acta Crystallogr.* A64, 112-122, https://doi.org/10.1107/S0108767307043930 (2008).

13. Sheldrick, G. M. Crystal structure refinement with SHELXL. *Acta Crystallogr.* C71, 3-8, https://doi.org/10.1107/S2053229614024218 (2015).

14. Dolomanov, O. V. et al. OLEX2: a complete structure solution, refinement and analysis program. *J. Appl. Crystallogr.* 42, 339-341, https://doi.org/10.1107/S0021889808042726 (2009).

15. TanabeSugano v. 1.4.1 (GitHub, 2023).

16. Fonger, W. H. & Struck, C. W. Temperature dependences of Cr^{3+} radiative and nonradiative transitions in ruby and emerald. *Phys. Rev. B* 11, 3251-3260, https://doi.org/10.1103/PhysRevB.11.3251 (1975).

17. Nelson, D. F. & Sturge, M. D. Relation between Absorption and Emission in the Region of the R Lines of Ruby. *Phys. Rev.* 137, A1117-A1130, https://doi.org/10.1103/PhysRev.137.A1117 (1965).

18. Zhang, Z. Y., Grattan, K. T. V. & Palmer, A. W. Temperature dependences of fluorescence lifetimes in Cr^{3+}-doped insulating crystals. *Phys. Rev. B: Condens. Matter* 48, 7772-7778, https://doi.org/10.1103/physrevb.48.7772 (1993).

19. Hu, Y. L. et al. Ruby-based decay-time thermometry: effect of probe size on extended measurement range (77–800 K). *Sens. Actuators, A-Phys* 63, 85-90, https://doi.org/10.1016/s0924-4247(97)01523-9 (1997).

20. Herzberg, G. Molecular Spectra and Molecular Structure. Volume I: Spectra of diatomic molecules. 2nd edn. New York: van Nostrand, 1963, 280-298 (https://archive.org/details/molecularspectra0001herz).

21. Struve, B. & Huber, G. The effect of the crystal field strength on the optical spectra of Cr^{3+} in gallium garnet laser crystals. *Appl. Phys. B Photophysics and Laser Chem.* 36, 195-201, https://doi.org/10.1007/bf00704574 (1985).

22. Wojtowicz, A. J., Grinberg, M. & Lempicki, A. The coupling of $^4T_2$ and ^2E states of the Cr^{3+} ion in solid state materials. *J. Lumin.* 50, 231-242, https://doi.org/10.1016/0022-2313(91)90047-y (1991).
